# RNA modifications in cellular metabolism: implications for metabolism-targeted therapy and immunotherapy

**DOI:** 10.1038/s41392-024-01777-5

**Published:** 2024-03-27

**Authors:** Wei-Wei Liu, Si-Qing Zheng, Tian Li, Yun-Fei Fei, Chen Wang, Shuang Zhang, Fei Wang, Guan-Min Jiang, Hao Wang

**Affiliations:** 1 Department of Laboratory Medicine, The First Affiliated Hospital of USTC, Division of Life Sciences and Medicine, University of Science and Technology of China, Hefei, China; 2 School of Clinical Medicine, Shandong University, Jinan, China; 3 Core Unit of National Clinical Research Center for Laboratory Medicine, Hefei, China; 4 Neurosurgical Department, The First Affiliated Hospital of USTC, Division of Life Sciences and Medicine, University of Science and Technology of China, Hefei, China; 5 Department of Clinical Laboratory, The Fifth Affiliated Hospital, Sun Yat-sen University, Zhuhai, China

**Keywords:** Senescence, Epigenetics

## Abstract

Cellular metabolism is an intricate network satisfying bioenergetic and biosynthesis requirements of cells. Relevant studies have been constantly making inroads in our understanding of pathophysiology, and inspiring development of therapeutics. As a crucial component of epigenetics at post-transcription level, RNA modification significantly determines RNA fates, further affecting various biological processes and cellular phenotypes. To be noted, immunometabolism defines the metabolic alterations occur on immune cells in different stages and immunological contexts. In this review, we characterize the distribution features, modifying mechanisms and biological functions of 8 RNA modifications, including N6-methyladenosine (m6A), N6,2′-O-dimethyladenosine (m6Am), N1-methyladenosine (m1A), 5-methylcytosine (m5C), N4-acetylcytosine (ac4C), N7-methylguanosine (m7G), Pseudouridine (Ψ), adenosine-to-inosine (A-to-I) editing, which are relatively the most studied types. Then regulatory roles of these RNA modification on metabolism in diverse health and disease contexts are comprehensively described, categorized as glucose, lipid, amino acid, and mitochondrial metabolism. And we highlight the regulation of RNA modifications on immunometabolism, further influencing immune responses. Above all, we provide a thorough discussion about clinical implications of RNA modification in metabolism-targeted therapy and immunotherapy, progression of RNA modification-targeted agents, and its potential in RNA-targeted therapeutics. Eventually, we give legitimate perspectives for future researches in this field from methodological requirements, mechanistic insights, to therapeutic applications.

## Introduction

Since the first documentation of RNA modification as early as 1950s, over 170 types have been identified, ubiquitously existing in coding RNAs and non-coding RNAs.^1^ The contributions of nucleoside base modifications in developing mRNA vaccine against COVID-19, which is awarded the Nobel Prize for 2023, immensely refresh the biologists studying RNA-based therapeutics. In most situations, the RNA modifications we talked about are reversible type, similar to DNA methylation. These modifications are deposited, removed and recognized by dedicated machineries, composed of writers, erasers and readers. These post-transcriptional modifications alter the canonical ribose and base structure to determine RNA fates, including splicing, trafficking, degradation, translation, and so on. Via regulating gene expression and cellular phenotypes, RNA modifications are extensively involved in various cellular processes.^2^

Cellular metabolism, a sophisticated network involving multitudes of biochemical reactions, continuously invigorates scientific researches. “Metabolism reprogramming” was originally proposed in cancer research, and gradually expanded to other non-tumor diseases and normal physiological processes. Used to be defined as “changes of tumor cellular bioenergetics”, the current perception tends to regard it as an inherent adaptive capacity of all cells, which is strengthened in tumor cells via abnormally activated pre-existing processes.^3^ Such metabolic adaptability is based on the interaction between cells and environment. During these biochemical processes, epigenetic modifications adjust the cell-environment relationship in a context-dependent manner.

According to the alarming statistics of several recent public health researches, metabolic diseases appear as an increasingly severe burden in human society. There have been more than 1.9 billion adults and over 650 million adults qualified as obese and overweight globally in 2016.^4^ According to International Diabetes Federation, 537 million adults had diabetes in 2021, which has become the ninth major cause of death worldwide.^5^ NAFLD is the most common chronic liver disease worldwide, the global prevalence of which was 25%.^6^ On the other hand, recent clinical trials targeting cancer metabolism come out with unsatisfactory efficacy and frustrating adverse reactions.^7–9^ Leaving dietary interventions alone, metabolic therapy is divided into agents targeting nucleotide metabolism and non-nucleotide metabolism. Not a few metabolic drugs targeting nucleotide metabolism, mostly nucleotide analogs, have been commonly employed in clinical practice. But development of the non-nucleotide metabolism-targeted drug remains in its nascent stages.

Therefore, for better insights into pathophysiology and optimized therapeutic strategies, integrated multi-omics analysis of metabolism is imperative. Not a few excellent studies have discussed the regulatory roles of RNA modifications on metabolism in specified pathological situations, with a particular focus on cancers.^10–12^ However, there is a deficiency of a comprehensive and wide-scale review on the epigenetic-metabolic interaction covering health and disease context.

Notably, immunometabolism is emerging field, expected to provide novel therapeutic strategies in cancer, autoimmunity, and metabolic diseases. The concept illustrates metabolic changes occurring in immune cells during their differentiation and activation processes. Studies have confirmed that various immune cells, including T cells, macrophages, NK cells, and DCs, proceeded metabolism remodeling to fulfill their specific functions in discrete contexts.^13–16^ According to current knowledge, RNA modification could exert influences on immunometabolism through cell-intrinsic and extrinsic mechanisms. The former is intrinsic programs, including mTORC1 signaling and metabolic-related genes expression. The latter refers to tissue microenvironment and nutrient availability.

In this review, we first introduce the history and current understanding of RNA modifications, and focus on their regulatory roles in cellular metabolism to construct epigenetic-metabolic landscape in physiological and pathological situations. And the influences of RNA modifications on immunometabolism in different immune responses is discussed separately. Eventually, we highlight the clinical implications of RNA modifications and provide perspectives for further studies.

## Overview of RNA modifications

### Brief history of RNA modification research

**Fig. 1:**
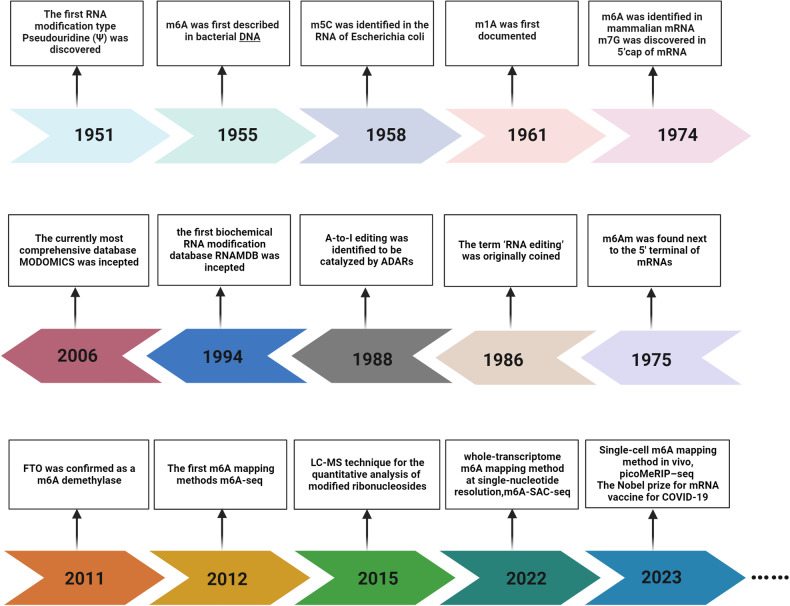
The milestone events in RNA modification field. The first RNA modification type Pseudouridine (Ψ) was discovered in 1951. Since then, other RNA modification, including m6A, m1A, m6Am, etc were discovered. Along with the accumulation of epi-transcriptomic knowledge, comprehensive databases like RNAMDB and MODOMICS were incepted. Since Liquid Chromatograph-Mass Spectrometer (LC-MS) technique was utilized for the quantitative analysis of modified ribonucleosides in 2015, more specific high-throughput mapping methods gradually emerged. Recent years have witnessed the application of single-cell sequencing technologies in mapping RNA modification. The figure is generated with BioRender (https://biorender.com)

Modified nucleosides in RNA, beyond the canonical A, U, C and G, have been recognized for more than half a century. Figure 1 illustrates the historical milestones of RNA modifications research. Pseudouridine (Ψ) is the first RNA modification type to be identified in 1950s.^17^ In 1965, sequencing of the first biological RNA, alanine tRNA derived from yeast, confirmed 10 modification types.^18^ Due to technological advancement, over 170 RNA modifications have been discovered, ubiquitously existing in various coding and non-coding RNAs. However, it was not until last decade that the functional significance of RNA modifications gradually got recognized, prominently the widespread prevalence and biological functions of N6-methyladenosine (m6A).^19^ Following 5′ cap and 3′ poly(A) tail of messenger RNA (mRNA), internal modifications on mRNAs were identified, represented by the most common methylation m6A.^20,21^ These modifications were observed to exert significant roles in every link of mRNA fate, including pre-mRNA splicing, nuclear export, translation, stabilization and degradation. Transfer RNA (tRNA) modification is renowned for the largest number, with an average of 13 modifications per molecule.^22^ Their biological roles could be generalized in two aspects, which are maintaining the tertiary structure and facilitating codon–anticodon recognition.^23^ For ribosomal RNA (rRNA), RNA modifications are especially indispensable, as rRNA biogenesis is interrupted without pseudouridines and 2′-*O*-methyls. Modifications of long noncoding RNA (lncRNA) are mainly methyl nucleotide derivatives, including m6A and m5C.^24^ Though far from being elucidated, lncRNAs modifications have been revealed to influence the stability, protein interactions, and subcellular distribution of lncRNAs.^25^ Human small nuclear RNA (snRNA) contains 2′-*O*-methyls, pseudouridines, and base methylations, participating in RNA splicing reaction. At present, mainstream RNA-seq methods are incapable for comprehensive and quantitative mapping of modifications on small non-coding RNAs. Here we summarize the current knowledge of RNA modifications, focusing on the regulatory mechanisms and biological consequences of several well-learned types.

### Main types of RNA modification

**Fig. 2:**
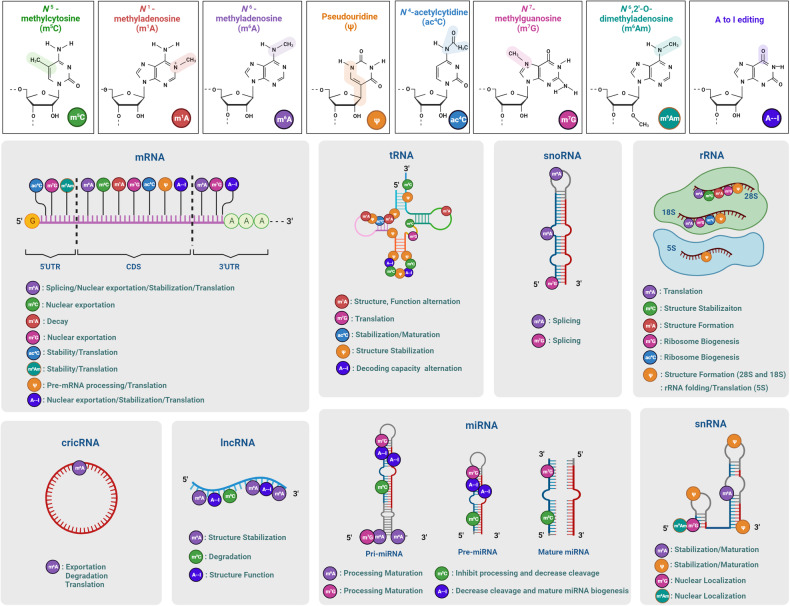
The chemical structure, distribution, and molecular functions of eight RNA modifications. Chemical modification occurs on many types of RNA and modulate every links of RNA metabolism. m6A N6-methyladenosine, m6Am N6,2′-O-dimethyladenosine, m5C 5-methylcytosine, m1A N1-methyladenosine, m7G 7-methylguanosine, ac4C N4-acetylcytidine, ψ pseudouridine, A-to-I editing adenosine-to-inosine RNA editing, CDS coding sequence, UTR untranslated regions, pri-miRNA primary microRNA, pre-miRNA precursor microRNA. The figure is generated with BioRender (https://biorender.com)

To build a general intuition of RNA modifications, we first sketch the distinctions between reversible and non-reversible modifications. Reversible types are usually smaller-scale modifications on chemical side chains, spanning from simple methylation to some appendages of large-molecular mass. These plastic and reversible RNA modifications extensively exist in gene regulation and cellular states. The extensive catalog of nonreversible RNA modifications includes RNA editing, splicing, and transcript-content modification (such as intron retention). Contrary to the reversible type, these modifications directly alter the sequence information, magnifying plasticity and diversity of transcriptome. The chemical structure and distribution of eight RNA modifications were showed in Fig. 2 and Table 1.

**Table 1:** The general characteristics of main RNA modification types

Type	Contribution	Class	Regulator	Function	Ref.
m6A	mRNA, rRNA, snRNA, snoRNA, miRNA, lncRNA, circRNA, eRNA	writer	METTL3	Catalyzes most of the m6A modifications via forming methyltransferase complex (MTC) with METTL14	^34^
			METTL14	Provides structural support in MTC	^35^
			METTL16	Catalyzes m6A in U6 snRNA	^40^
		eraser	FTO	Removes m6A/m6Am/m1A modifications	^44,45^
			ALKBH5	Demethylates m6A modification exclusively	^50^
		reader	YTHDF1	Stabilizes transcripts and initiates translation	^56,57^
			YTHDF2	Promotes degradation	^56,57^
			YTHDF3	Facilitates translation and degradation	^56,57^
			YTHDC1	Mediates RNA splicing, nuclear export and degradation	^54^
			YTHDC2	Promotes translation efficacy and decay	^55^
			IGF2BP1/2/3	Stabilizes transcripts and facilitates translation	^58,59^
			HNRNPs	Mediate splicing of pre-mRNAs and/or pri-miRNAs	^60^
m6Am	mRNA, snRNA	writer	PCIF1	Catalyzes m6Am next to the 5′ cap of mRNAs and in snRNAs	^77^
			METTL4	Catalyzes m6Am at position 30 in human U2 snRNA	^80^
		eraser	FTO	Removes m6A/m6Am/m1A modifications	^49^
m1A	tRNA, rRNA, lncRNA, and mRNA	writer	TRMT61B	Catalyzes mA at positions 58 (m1A58)	^92^
			TRMT10C	Catalyzes mA at positions 9 (m1A9)	^92,93^
			TRMT6/61A	Catalyzes m1A in tRNA at A58 and mRNA	^94^
			TRMT61B	Mediates m1A in mitochondrial 16S rRNA	^95^
		eraser	ALKBH1	Catalyzes demethylation of most m1A in cyto-tRNAs	^97^
			ALKBH3	Demethylates m1A in both tRNAs and mRNAs	^98,99^
			ALKBH7	Demethylates m1A within mitochondrial Leu1 pre-tRNA regions	^100^
			FTO	Demethylates m1A in tRNA	^49^
		reader	YTHDF1/2/3 YTHDC1	Mediates stabilization, degradation, splicing, translation	^101,102^
m5C		writer	NSUN1	Catalyzes m5C at position 4413 of 28S rRNA	^114^
			NSUN5	Catalyzes m5C at position 3761 of 28S rRNA	^115^
			NSUN2	Methylates C34, C40, C48, C49, and C50 in several tRNAs	^116^
			NSUN6	Methylates C72 in particular tRNAs	^117^
			DNMT2	Methylates C38 in particular tRNAs	^118^
			NSUN3	Catalyzes m5C in mitochondrial tRNA	^119^
			NSUN4	Catalyzes m5C in 12S rRNA	^120^
		eraser	ALKBH1	Demethylates m5C at position 34 of cytoplasmic and mitochondrial tRNA	^126,127^
			TET1/2/3	Catalyzes first step of m5C demethylation	^128^
		reader	ALYREF	Promotes the nuclear export of m5C-modified rRNAs	^129^
			YTHDF2	Modulates the maturation of m5C-modified rRNAs	^130^
**ac4C**	mRNA, tRNA, rRNA	writer	NAT10	THUMPD1 and snoRNP are necessary assistants for modifying tRNA and 18S rRNA, respectively	^141,140,141^
**m7G**	mRNA, tRNA, rRNA, miRNA	writer	METTL1	Forms complex with WDR4 to catalyze m7G on tRNA, miRNA, and mRNA	^147^
			RNMT	Catalyzes m7G on recapped mRNAs, cooperated with RAM	^149^
			WBSCR22	methylate G1639 in human 18S rRNA, cooperated with TRMT112	^150^
			TGS1	Catalyzes hypermethylation of m7G caps into m2,2,7G in snRNAs and snoRNAs	^151^
		reader	eIF4E, CBC	recognizes m7G cap and further affect RNA maturation, nuclear export, and translation	^152^
**Ψ**		writer	DKC1	Forms a complex with box H/ACA snRNA to pseudouridylates rRNA	^160^
**A-to-I**	Pre-mRNA, pri-miRNA	writer	ADAR1-3	ADAR1 and ADAR2 could catalyze all known A-to-I editing events, while ADAR3 has no deaminase activity	^186^

#### Adenosine modification

##### N6-methyladenosine (m6A)

m6A modification refers to the methylation of the adenosine base at the N-6 position. m6A targeted at consensus sequences DRACH (D = G, A, or U; R = G or A; H = A, C, or U), which are mainly enriched in CDS and 3’UTR region of mRNA,^26^ as well as most non-coding RNAs, including rRNAs, lncRNAs, circular RNAs (circRNAs), microRNAs (miRNAs), small nuclear RNAs (snRNAs), small nucleolar RNAs (snoRNAs).^27^ Growing studies have confirmed that m6A could exert significant impacts on various biological processes in mammals, including DNA damage response, cell cycle, circadian rhythm, heat shock response, meiotic progression, development of hematopoietic, central nervous and reproductive systems, myogenesis, and fat differentiation.^28–33^

m6A deposition in mRNA is dependent on methyltransferase complex (MTC), of which the methyltransferase-like 3/14 (METTL3/14) heterodimer is the key component.^34^ Therein, METTL3 exerts catalytic role via transferring methyl group of S-adenosyl methionine (SAM) and METTL14 provides structural support. In METTL3, two methyltransferase domains (MTD) bind to methyl donors, CCH-type zinc finger domain (ZFD) recognizes targets, while nuclear localization signal (NLS) domain and leading helix structure (LH) domain coordinately mediate the interaction between METTL3 and METTL4.^35,36^ There are several auxiliary subunits for localizing and initiating methylation, including Wilms’ tumor 1-associating protein (WTAP), RNA-binding motif protein 15/15B (RBM15/15B), zinc finger CCCH-type containing 13 (ZC3H13) and vir-like m^6^A methyltransferase-associated (VIRMA, also known as KIAA1429).^37–39^ METTL16 is responsible for m6A formation in U6 snRNA, targeting a conserved UACAGAGAA sequence.^40^ METTL16 also participates in maintaining homeostasis of SAM in a m6A-dependent manner.^41^

Zinc Finger CCHC-Type Containing 4 (ZCCHC4) and METTL5 mediate m6A modification of 28 S and 18 S rRNA at A4220 and A1832 region, respectively.^42,43^ Both m6A demethylases, Fat mass and obesity-associated protein (FTO) and AlkB homolog 5 (ALKBH5), belong to AlkB family of the Fe (II)/α-ketoglutarate-dependent dioxygenase superfamily. FTO is the first discovered m6A eraser for mRNA and snRNA, which also mediates demethylation of N6,2′-O-dimethyladenosine (m6Am) and N1-methyladenosine (m1A).^44,45^ Which one of m6A and m6Am is the principal substrate of FTO remains controversial. It was reported that FTO catalyzed m6A demethylation at a concentration at least twice that of m6Am.^46,47^ But Zhang et al. proposed that FTO equivalently demethylated m6A and m6Am deposited on the same RNA sequence.^48^ Significantly, Wei et al. discovered that nuclear FTO showed an affinity bias towards m6A, which tends to be inconspicuous in cytoplasm, due to altered abundance of m6A.^49^ Whereas, ALKBH5 exclusively catalyzes m6A demethylation in mRNA.^50^

The most studied readers are the YT521-B homology (YTH) domain family members, including YTHDF1/2/3 and YTHDC1/2, most of which localize to cytoplasm except for YTHDC1 in nucleus.^51,52^ The prevailing idea is that YTHDFs bind to different m6A-modified RNAs, but Zaccara et al. hold that all m6A-modified RNAs are subjected to YTHDFs and they act redundantly in mediating RNA degradation.^53^ YTHDC1 contributes to RNA splicing, nuclear export and degradation, while YTHDC2 promotes translation efficacy and decay.^54,55^ YTHDF1 could stabilize transcripts and initiate translation via interacting with eIF3, YTHDF3 not only facilitates translation but works in synergism with YTHDF2 in inducing mRNA degradation.^56,57^ The insulin-like growth factor 2 mRNA-binding protein family, IGF2BP1/2/3, is another group of readers. IGF2BPs possess 4 repetitive KH domains and bind to m6A sites with KH3/4 to stabilize transcripts and facilitate translation.^58,59^ The heterogeneous nuclear ribonucleoprotein (HNRNP) family includes HNRNPC, HNRNPG, and HNRNPA2B1. HNRNPs can mediate splicing of precursor (pre)-mRNAs and/or primary (pri)-miRNAs through ‘the m6A-switch’ mechanism, in which m6A alters the local structure of mRNA or lncRNA to facilitate the binding of HNRNPs.^60^ HNRNPA2B1 directly binds to pri-miRNAs to mediate alternative splicing. Meanwhile, its interaction with the miRNA microprocessor complex protein DGCR8 promoted primary miRNA processing.^61^ And HNRNPG could elicit co-transcriptional m6A-dependent alternative splicing regulation via directly binding to RNA polymerase II (RNAPII).^62^ Besides, proline rich coiled-coil 2 A (PRRC2A) and Staphylococcal nuclease and tudor domain-containing 1 (SND1) could serve as readers to stabilize m6A-modified RNAs.^63,64^

To sum up, m6A modification extensively influences fate of different RNA classes, consequently regulates various cellular processes. In mRNAs, m6A methylation can affect splicing, exportation, stabilization, degradation, and translation.^65^ In rRNAs, the A1832 methylation in 18 S rRNA and A4220 methylation in 28 S rRNA are essential for translation.^42,43^ In miRNAs, m6A could facilitate pri-miRNA processing via recruiting DGCR8,^61^ or downregulate several miRNAs via some exclusive mechanism.^66^ In lncRNAs, m6A modification could serve as a structural switch to regulate RNA-protein interactions,^67^ or stabilize lncRNAs to ensure its function.^68^ In cirRNAs, m6A could facilitate cytoplasmic export,^69^ translation^70^ and degradation.^71^ Moreover, m6A participates in modulating splicing and biogenesis of snRNA.^72^ Although m6A methylation has been widely investigated, the underlying rationales are far from clarified. For example, m6A modification could modulate RNA life via diverse mechanisms, but how these selective effects are determined in different cellular contexts remains unclear. While previous studies notably focus in mRNAs, the interplay between m6A and non-coding RNAs deserves more attention. The same is true for m6A readers, which are unheeded compared to writers and erasers. And the significance of methodology development cannot be stressed enough, as bona fide m6A mapping and elaborate edition on specific m6A sites will provide a wide scope for future researches.

##### N6,2′-O-dimethyladenosine (m6Am)

m6Am is produced at a 2′-O-methylated adenosine which is methylated co-transcriptionally at the N6 position. It is discovered in the first position adjacent to the 5′ cap structure in many mRNAs and snRNAs in mammals, and also found as internal modification in the snRNA U2.^73^ According to quantification studies, m6Am content ranges from 10% to almost 50% in mRNAs of different organisms and cell types.^74^ Previous studies have shown that m6Am installed by host PCIF1 on viral RNA mediated immune evasion, while host m6Am exhibited both anti-viral and pro-viral roles.^75,76^

The enzyme catalyzes m6Am next to the 5′ cap of mRNAs and in snRNAs is “phosphorylated CTD-interacting factor 1” (PCIF1), also known as “cap-specific adenosine methyltransferase” (CAPAM).^77^ The core region of PCIF1 contains the methyltransferase domain and helical domain that functions as the RNA-binding surface,^78^ and a specific site (m7Gsite) located between the two domains mediated the specific recognition of the m7G cap.^79^ It was revealed that knockout of PCIF1 altered cell proliferation under oxidative stress conditions in human HEK293T cell line.^79^ Another m6Am writer, METTL4 methylated the internal 2′-O methylated adenine, at position 30 in human U2 snRNA.^80^ METTL4 contains a C-terminal domain that is similar to METTL3, a middle domain (MID) and a N-terminal domain (NTD), which enables METTL4 works as a monomer with no need for METTL14.^81^ It was indicated that METTL4 was highly conserved and exclusive for U2 snRNA.^82^ However, overexpressed METTL4 tends to modify A instead of Am in mRNAs with consensus HMAGKD (H = A/C/U, M = A/C, K = G/U, D = A/G/U).^83^ Also, METTL4 was found to catalyze mt-DNA m6A in human cell line.^84^ Ablation of METTL4 did not influence viability of HEK293T cell line, but altered adipocyte differentiation of mouse 3T3-L1 cells.^83,85^

To date, FTO is the only known demethylase for m6Am, which, as mentioned above, show a substrate preference between m6A and m6Am depending on its cellular localization.^49^ In cytoplasm, FTO preferentially demethylates cap-adjacent m6Am and internal m6A on mRNAs, while nuclear FTO acts on m6Am in RNA Pol II-transcribed snRNAs, and internal m6Am and m6A in the snRNAs U2 and U6.^49^ Studies have identified that FTO distribution was correlated with cell cycle phase and regulated by casein kinase II-mediated phosphorylation.^86^ To be mentioned, structural analysis demonstrated that the catalytic activity of FTO was mediated by recognizing N6-methyl of adenine rather than the 2′-*O* methyl group of the ribose.^48^

There are discrepancies exist in present studies on influences of m6Am modification on gene expression, as an inherited issue from the past immature m6Am mapping methodologies. For instance, m6Am methylation was initially suggested to play a positive role in mRNA stability in a cell-type-specific manner.^45^ However, a recent study, developing the specific sequencing method m6Am-seq, has clarified that PCIF1 was not required for stabilization of m6Am-modified mRNAs.^77^ There are other studies implicated that m6Am did not have direct effects on mRNA stability.^77,79^ As for translation, the current cognition is that m6Am modifications in mRNA cap exert a cell-specific influence on translation.^79,87^ And such effects are dependent on 2′-O-methylation modification in the second nucleotide of the cap-structure.^88^ Moreover, the effect of m6Am modification in splicing need more verification. It was suggested that METTL4 had no direct influence on U2 snRNA expression levels but rather altered splicing regulation.^80,82^

To sum up, the cap-adjacent location endows m6Am modification with potential to regulate stability and translation. The significant discovery of PCIF1, specifically catalyzes m6Am in the cap structure, drives relevant exploration. However, methodological deficiency is the major problems in m6Am researches. Most of previous studies adopted m6A mapping protocols instead of specific m6Am mapping methods, which led to poor reproducibility and controversial results. Thus, more specific and efficient methods are in urgent need to clarify the regulatory roles of m6Am modification in gene expression.

##### N1-methyladenosine (m1A)

m1A, the methylation of adenosine at position N1 identified in 1960s, has been found in tRNA, rRNA, lncRNA, and mRNA, among which tRNA is the most heavy-modified class.^89,90^ Particularly, m1A can transfer to m6A after “Dimroth rearrangement” under alkaline conditions and they also share some regulators.^49^ m1A has been identified and enriched in specific regions of viral RNA, but its influences in innate immunity is not yet clear.^91^

In mitochondrial tRNA, m1A methylation is catalyzed by tRNA methyltransferase (TRMT61B) and TRMT10C at positions 58 (m1A58) and 9 (m1A9), respectively.^92,93^ TRMT61A and TRMT6 form a heterotetrameric complex to methylate both cytoplasmic tRNA at A58 and mRNAs with GUUCRA tRNA-like motifs, as TRMT61A functions as the catalytic subunit.^94^ TRMT61B mediates m1A in mitochondrial 16 S rRNA,^95^ and nucleomethylin (NML, also known as RRP8) methylates 28 S rRNA in nuclei.^96^ And no specific m1A writer for mRNA has been reported yet. ALKBH1 catalyzes demethylation of most m1A in cyto-tRNAs, while m1A58 is the major substrate.^97^ ALKBH3 demethylates m1A in both tRNAs and mRNAs.^98,99^ ALKBH7 can demethylate m1A within mitochondrial Leu1 pre-tRNA regions in the nascent polycistronic mitochondrial RNAs.^100^ And FTO was also proved to demethylated m1A in tRNA.^49^ YTHDF1/2/3 and YTHDC1 have been confirmed to directly bind to m1A marks, with weaker affinity than that of m6A.^101,102^ The evolutionarily conversed YTH domain was suggested to be the key to methyl recognition, but the mechanistic research remains deficient.^102^

The methyl group of m1A carries a positive electrostatic charge, which affects RNA base pairing, and subsequently influences molecule structure and function of modified RNAs. Notably, the electro-chemical interaction of m1A is supposed to play roles in maintaining or stabilizing the *T*-loop-like structure, and further strengthening the structure.^103^ As for translation, m1A modification has effects on initiation or elongation process via regulating tRNA, mRNA and rRNA. Several studies have indicated that m1A on either tRNA or mt-tRNA could facilitate translation.^97,104^ Whereas, m1A modification on mRNA plays diverse roles in protein synthesis, as m1A in 5’UTR correlates with enhanced translation initiation and efficiency,^105^ but m1A in the CDS exerts inhibitory effects.^92,106^ In rRNA, m1A is likely associated with translation initiation, as loss of yeast RRP8-catalyzed m1A led to incompetent formation of the 80 S initiation complex.^107^ Moreover, m1A modification participates in the structural thermostability of tRNAs^108^ and the nascent polycistronic mt-RNA processing.^100^

As one of the most abundant internal RNA modifications, the machinery and biological functions of m1A remain largely unknown. The roles of YTH domain-containing proteins as m1A readers may provide novel scientific prospects. And whether its impact on RNA base pairing influences RNA interaction, such as miRNA with mRNA, lncRNA, and circRNA, requires more exploration.

#### Cytosine modification

##### 5-methylcytosine (m5C)

For decades, methylation of cytosine residues at the position 5 in DNA have been quite familiar. Ever since it was identified in RNA in 1958, m5C has been revealed to distribute widely in RNAs, including tRNA, rRNA, mRNA, enhancer RNA (eRNA), and miRNA.^109,110^ Studies figured out that m5C modification extensively occurred on maternal mRNA in zygotes of different eukaryotic species, regulating embryogenesis in mouse, zebrafish and Drosophila.^111–113^

In eukaryotes, m5C modification is catalyzed by members of the NOL1/NOP2/SUN domain (NSUN) family of proteins, NSUN1-7 and DNA methyltransferase (DNMT) homolog DNMT2. For rRNA, NSUN1 and NSUN5 introduce m5C at position 4413 and 3761 of human 28 S rRNA, while their homologs in yeast methylate 25S-C2870/25S-C2278.^114,115^ For tRNA, NSUN2 could modify several sites in various tRNAs, including C34, C40, C48, C49, and C50.^116^ NSUN6 and DNMT2 methylate C72 and C38 in particular tRNAs, respectively.^117,118^ NSUN3 and NSUN4 are responsible for methylation of mitochondrial tRNA and 12 S rRNA.^119,120^ And NSUN4 forms a complex with the mitochondrial transcription factor MTERF4 for lack of RNA recognition motif.^121^ The m5C methyltransferase specific for mRNAs has not been confirmed yet, but NSUN2 was described to target mRNAs in several studies.^122,123^ Besides, m5C modifications of ncRNA and eRNA are modified by NSUN2 and NSUN7, respectively.^124,125^ The identified m5C erasers include ten-eleven translocation (TET) proteins (TET1–3) and ALKBH1. ALKBH1 can successively catalyze m5C into 5-hydroxymethylcytidine (hm5C), 5-formylcytosine (f5C), and 5-carboxylcytosine, at position 34 of cytoplasmic and mitochondrial tRNA,^126,127^ whereas TETs has been only reported to complete the first step for RNA m5C.^128^ Aly/REF Export Factor (ALYREF) is the first identified m5C reader in mRNA, a well-known complex that promotes the nuclear export.^129^ Y-box-binding protein 1 (YBX1) is located in cytoplasm and could recruit stability maintainer ELAV like RNA binding protein 1 (ELAVL1) to stabilize m5C-modified mRNAs.^112^ Also, YTHDF2 has been reported to modulate the maturation of m5C-modified rRNAs.^130^

Collectively, m5C modification plays a crucial role in RNA stabilization, exportation, and translation. m5C at C2278 of 25 S rRNA stabilizes the structural conformation of the ribosome.^115^ Hypermethylated mRNAs with m5C are stabilized via YBX1-dependent manner.^131^ NSUN2-mediated m5C modifications in vault RNA are significant for its processing into derived small RNAs and protect eRNAs from degradation.^132^ Also, NSUN2 modified cyclin-dependent kinase inhibitor 1 A (CDKN1A) mRNA and promoted its nuclear export and translation.^133^

As mentioned above, the dizzying matchup between m5C modifiers and their specific targets brings out challenges as well as opportunities. Targeting certain writers or manipulating specific modification sites reserve great therapeutic potential.

##### N4-acetylcytosine (ac4C)

ac4C, acetylation of the N4 position of cytosine, is the first acetylation event described. As initially found in tRNA and rRNA, ac4C was also confirmed to be widely present on mRNAs.^134^ In tRNA, ac4C is located at the wobble of tRNA^Met^ and the D-arm of tRNA^Ser/Leu^.^135^ In eukaryotic 18 S rRNA, ac4C is deposited in helix 34 and helix 45 near the decoding site.^136^ In mRNA, ac4C is detected in the CDS region and 5ʹUTR, enriched in the third codon encoding amino acid.^134^ Advances in the study of RNA ac4C modification in cell cycle, inflammatory stress, tumors, premature diseases and viral infection have been reported.^91,137,138^

Currently, N-acetyltransferase 10 (NAT10) is the only identified ac4C writer, with acetyl-CoA providing acetyl and ATP/GTP hydrolysis supplying energy.^139^ When modifying tRNA, the assistance of THUMP domain containing 1 (THUMPD1) is necessary,^140^ while box C/D snoRNPs act as antisense to guide 18 S rRNA acetylation.^141^ For now, no ac4C eraser has been identified and it remains unknown whether ac4C modification is reversible.

The presence of ac4C on tRNA helps maintain the thermal stability of tRNA and a high heat tolerance of cells, and improves fidelity and efficiency of translation.^134,142^ ac4C on mRNA CDS region significantly enhance mRNA stability and facilitate translation, probably by preserving codon-anticodon interaction.^143^ However, ac4C on 5ʹUTR mainly regulates translation initiation in a location-specific manner, as ac4C downstream a weak translation initiation site could promote translation, but the one adjacent to a strong AUG start codon disturbs translation.^144^ In 18 S rRNA, ac4C modification is crucial for maintaining translation accuracy, pre-rRNA processing and ribosome synthesis.^140^

The cognition of ac4C modifiers and molecular functions remains largely unknown. Since cofactors of NAT10 have been identified during ac4C formation in human rRNA or tRNA, whether novel cofactors exist in catalyzing mRNA ac4C is noteworthy. Particularly, no erasers or readers has been found yet, whether a deacetylation mechanism exist require more validation.

#### Guanosine modification

##### N7-methylguanosine

m7G, referring to the RNA methylation of guanine at position N7, was first found at the 5′ cap (m7GPPPN) of mRNA, stabilizing transcripts and further mediating cap-related biological functions.^145^ Until now, m7G has been discovered at internal position within mRNA, tRNA, and rRNA,^146,147^ and tRNA nucleotide position 46 (m7G46) in the variable loop region is the most prevalent m7G methylation site.^148^

The most well-characterized m7G writer is METTL1, which forms a functional complex with WD repeat domain 4 (WDR4) to install m7G on tRNA, miRNA, and mRNA.^147^ RNA guanine-7 methyltransferase (RNMT) is responsible for m7G on recapped mRNAs, cooperated with RNMT-activating mini-protein (RAM).^149^ Williams–Beuren syndrome chromosome region 22 (WBSCR22) methylate G1639 in human 18 S rRNA, requiring tRNA methyltransferase activator subunit 112 (TRMT112).^150^ Trimethylguanosine synthase 1 (TGS1) might also function as a modifier, catalyzing hypermethylation of m7G caps into m2,2,7 G in snRNAs and snoRNAs.^151^ The eukaryotic translation initiation factor eIF4E and the cap-binding complex (CBC) can recognize m7G cap and further affect RNA maturation, nuclear export, and translation.^152^

Notably, m7G modification is extensively involved in various biological processes. For mRNA, the m7G cap could regulate pre-mRNA slicing, nuclear export, translation,^152^ and indirectly enhance translational capacity by driving ribosome biogenesis.^153^ And internal m7G also influences translation.^154^ For tRNA, METTL1/WDR4-mediated m7G methylome plays pivotal roles in maintaining tRNA structural integrity, thereby facilitating translation and reducing ribosome pausing.^155^ For rRNA, m7G modification participates in 18 S rRNA precursor biogenesis and nuclear export of the 40 S rRNA.^150,156^ Moreover, m7G on G-quadruplex structures in pri-miRNA could promote miRNA processing.^157^

At present, our understanding of m7G regulators is apparently limited. No specific demethylase has been identified to regulate the global balance of m7G. And whether m7G modification regulating gene expression via affecting the secondary structure of RNA or recruiting RNA binding proteins remains unclear. Furthermore, the interplay among m7G and other post-transcription attracts growing attention, more explorations are imperative to unravel the underlying mechanism.

#### Uridine modification

##### Pseudouridine (Ψ)

Ψ, the 5–riboside isomer of uridine, is the first discovered and most abundant RNA modification.^17,158^ The C5 atom, instead of N1, forms a new carbon-carbon bond (C5–C1′) with pentose at its non-Watson-Crick edge, endowing Ψ with unique chemical properties. Ψ is present in a wide range of RNAs, including tRNA, rRNA, and various snRNAs, which is highly conserved among species.^158,159^ The widespread distribution determines its importance in regulating gene expression, steering cellular programs both in development and disease.

The pseudouridylation is mainly catalyzed by pseudouridine synthases (PUSs), via RNA-dependent or -independent manner. The RNA-dependent mechanism involves Dyskerin pseudouridine synthase 1 (DKC1), which forms a complex with box H/ACA snRNA to pseudouridylates rRNA.^160^ The RNA-independent PUSs includes PUS1, PUSL1, PUS3, TRUB1, TRUB2, PUS7, PUS7L, RPUSD1–4, and PUS10.^161–163^ Regrettably, no Ψ eraser or reader has been documented. And it was speculated that C5–C1′ bond render pseudouridylation irreversible.^164^

Ψ on tRNA is critical for stabilizing tRNA structure and tRNA codon–anticodon base pairing, further affecting translation processes. Also, Ψ-modified tRNA-derived fragments could restrain aberrant protein synthesis.^165^ Besides, Ψ is also involved in pre-mRNA processing, structure and stability of mRNA, translational fidelity and termination.^166,167^ The rRNA Ψ plays a functional role in rRNA processing and protein synthesis.^168^ It was demonstrated that hypo-pseudouridylated rRNAs decreased affinity for tRNA of ribosomes, impairing translational fidelity.^169^ snRNP Ψ participates in its biogenesis and splicing.^170^ Ψ35 in the 5′ end of the U2 snRNA was considered as necessary for early spliceosome formation.^171^

Although discovered 70 years ago, there are still plenty of vacancies in knowledge on the mechanisms and functions of Ψ. Elucidating whether pseudouridylation is reversible will be one of the key directions in the future. Since efforts to approach inducible pseudouridylation have generated exciting results, which open up new avenues for exploring potential therapeutics. Remarkably, Ψ has already been validated to make critical contribution to COVID-19 mRNA vaccines.^172^

#### RNA editing

##### A-to-I editing

RNA editing modifies primary mRNA and miRNA in posttranscriptional level, altering coding information of DNA. It was first discovered in trypanosome mitochondrial mRNA in 1986.^173^ So far, RNA editing has been found in tRNA, rRNA and miRNA.^174–176^ The most prevalent type is conversion of adenosine into inosine (A-to-I editing),^177^ and then inosine is recognized as guanine by the translational machinery. It has been implicated that ADAR1-mediated A-to-I editing was involved in stem cell pluripotency and maintenance, neurological development and function, and immune response.^178,179^

A-to-I editing only occurs in the double-stranded regions of RNAs made from inverted Alu repetitive elements (Alu dsRNAs), and is far less frequent in coding sequences than noncoding sequences such as UTRs and introns.^180^ Precursors of certain miRNAs are also common targets.^181^ The editing levels dramatically vary in cell and tissue type of different origins and development stages, ranging from 2%-100%.^182,183^ The conversion is catalyzed by adenosine deaminase acting on RNA (ADAR) protein.^184^ In vertebrates, the isoforms of ADAR protein, ADAR1-3 have identified. These ADAR enzymes possess a C-terminal conserved catalytic deaminase domain, and double-stranded RNA binding domain (dsRBD) at the N-terminus, three for ADAR1 and two for ADAR2-3.^185^ Functionally, ADAR1 and ADAR2 are responsible for all known A-to-I editing events, while ADAR3 has no documented deaminase activity.^186^ The mechanism of ADAR substrate specificity remains unclear, in which length and structure of dsRNA was suggested to play an important role,^187^ and editor modulators like snoRNAs also participated in.^188^ The consequences of A-to-I editing in coding sequences includes alternative splicing, nonsynonymous amino acid substitutions, nuclear retention and degradation of mRNA. Also, these editing could regulate gene expression via influencing splicing enhancers/silencers recognition sites of ncRNAs in non-coding sequence.^189^ For several miRNAs, A-to-I editing negatively affects the expression and function of the mature miRNAs.^181^ In opposition, ADAR1 could facilitate miRNA processing and RNA interference (RNAi) efficacy via forming a complex with Dicer.^190^

Recently, RNA editing, represented by A-to-I editing, has emerged as a powerful tool to correct pathogenetic mutations, modulate gene expression and protein function. And its transient pharmacodynamic effects could be applied in treatment of several diseases like viral infection, obesity, inflammation, and acute pain. In addition, the transient modulation of protein functions opens up new avenues for oncology and regenerative drugs.

### Main database of RNA modifications

To our knowledge, there have been 15 databases established for RNA modifications, two of which are concentrated on biochemical features of RNA modifications, and the rest aimed at elucidating the biological roles. The latter part includes reversible RNA modification database, which can be further classified as comprehensive and type-specific, and nonreversible RNA modification database, namely RNA editing database (Table 2).

**Table 2:** Databases of RNA modifications

Name	Description	URL
**Biochemical RNA modification database**		
RNAMDB	A databse of basic chemical characterizations of 109 RNA modified nucleosides	https://mods.rna.albany.edu/
MODOMICS	the most comprehensive RNA modification pathway source	http://modomics.genesilico.pl
**Comprehensive reversible RNA modification databases**.		
m6A-Atlas	a comprehensive knowledgebase for unraveling the m6A epitranscriptome	www.xjtlu.edu.cn/biologicalsciences/atlas
m7GHub v2.0	a resource deciphering the location, regulation, and pathogenesis of internal mRNA m7G epitranscriptome	www.xjtlu.edu.cn/biologicalsciences/m7ghub
m5C-Atlas	a database for decoding and annotating the m5C epitranscriptome	https://www.xjtlu.edu.cn/biologicalsciences/m5c-atlas
MeT-DB v2.0	a database for investigation of m6A and its previous version is the first comprehensive resource for m6A in transcriptome	http://compgenomics.utsa.edu/MeTDB/
RMBase v2.0	a database deciphering the map of RNA modification from epitanscriptome sequencing data	http://rna.sysu.edu.cn/rmbase/
REPIC	an atlas of m6A methylome with cell lines or tissue specificity	https://repicmod.uchicago.edu/repic
**Specialized reversible RNA modification database**		
CVm6A	a visualization and exploration database for global m6A patterns across cell lines	http://gb.whu.edu.cn:8080/CVm6A
RMVar	a database of functional variants involved in RNA modifications	http://rmvar.renlab.org
RMDisease	a database unveiling the association between disease-associated variants and their epi-transcriptome disturbance	www.xjtlu.edu.cn/biologicalsciences/rmd
**RNA Editing Database**		
REDIdb	a specialized database for RNA editing modifications in plant organelles	http://srv00.recas.ba.infn.it/redidb/index.html
RADAR	a rigorously annotated database of A-to-I RNA editing in humans, mice and flies	http://RNAedit.com
DARNED	a repository for RNA editing in humans, centralized on A-to-I editing	https://darned.ucc.ie/
REDIportal	the largest and specialized repository for A-to-I editing occurring in a variety of human tissues	http://srv00.recas.ba.infn.it/atlas/

### Sequencing methods of RNA modification profiling

With the advances in next-generation sequencing (NGS) technologies, many experimental methods have been designed to profile RNA modifications. Generally, the principles of sequencing methods could be classified as two types. The first type is based on antibody or chemical label to capture modified RNA fragments, such as MeRIP-seq for m6A profiling. Another strategy is using enzyme-assisted reaction or a specific chemical reaction on the modified bases, such as Pseudo-seq for Ψ. And these reactions bring about base deletions, substitutions, or truncations, either before or after the modified bases. Here we briefly introduce characteristics of current sequencing methods in Table 3.

**Table 3:** Sequencing methods of RNA modifications

Technologies	Year	Resolution	Description	Ref.
MeRIP-seq (m6A-seq)	2012	100-200nt	m6A-specific sequencing method based on antibody-mediated capture and massively parallel sequencing	^442^
miCLIP	2015	single nucleotide	individual nucleotide resolution cross-linking and immunoprecipitation method for m6A and m6Am	^443^
PA-m6A-seq	2015	23nt	m6A sequencing assisted by photo-crosslinking	^444^
m6A-REF-seq	2019	single nucleotide	antibody-independent m6A mapping based on the m6A-sensitive RNA endoribonuclease	^445^
DART-seq	2019	single nucleotide	an antibody-free method for m6A targeting deamination adjacent to modification sites	^446^
m6ACE-seq	2019	single nucleotide	m6A cross-linking exonuclease sequencing method	^447^
m6A-SEAL-seq	2020	single nucleotide	FTO-assisted m6A selective chemical labeling method	^448^
m6A-label-seq	2020	single nucleotide	a metabolic labeling method for m6A	^449^
m1A-seq	2016	50-200nt	a protocol for mapping m1A at single-nucleotide resolution	^101^
m1A-ID-seq	2016	\	a m1A profiling method based on immunoprecipitation and the inherent ability of m1A to stall reverse transcription	^450^
m1A-MAP	2017	single nucleotide	a misincorporation- assisted profiling method for m1A	^451^
Aza-IP	2013	\	5-azacytidine-mediated RNAimmunoprecipitation	^452^
Bisulfite sequencing	2017	single nucleotide	a RNA bisulfite sequencing method of m5C	^453^
m5C-RIP-seq	2017	\	a m5C profiling using RNA immunoprecipitation followed by a deep sequencing	^454^
Pseudo-seq	2014	single nucleotide	a genome-wide, single-nucleotide-resolution method for pseudouridine	^167^
Ψ-seq/Psi-seq	2014	single nucleotide	a protocol for transcriptome-wide quantitative mapping of Ψ	^455^
CeU-seq	2015	single nucleotide	N3-CMC–enriched Ψ sequencing method	^456^
m7G-MeRIP-Seq	2019	100-200nt	m7G-methylated immunoprecipitation sequencing method	^154^
m7G-MaP-seq	2019	single nucleotide	high-throughput m7G mutational profiling sequencing	^457^
m7G-miCLIP-Seq	2019	single nucleotide	m7G individual-nucleotide-resolution cross-linking and immunoprecipitation with sequencing method	^458^
ac4C-RIP-seq	2018	\	transcriptome-wide ac4C-targeted RNA immunoprecipitation sequencing	^134^
ac4C-seq	2021	single nucleotide	a protocol for the quantitative single-nucleotide resolution mapping of ac4C	^459^
ICE-seq	2011	\	inosine chemical erasing method with deep sequencing method	^460^

## RNA modifications and cellular metabolism

Cellular metabolism is a flexible network that allows cells to satisfy their bioenergetic and biosynthesis requirements. In malignant cells, metabolism reprogramming is implicated in tumorigenesis, progression, metastasis and chemoresistance. Aside from the well-concerned cancer metabolism, metabolic adoptions extensively exist in various diseases, including diabetes, obesity, nonalcoholic fatty liver disease (NAFLD), and atherosclerosis. In these pathologies, dysregulated RNA modifiers significantly participate in metabolic alterations via targeting metabolic enzymes, transporters, metabolism-related transcription factors or pathways. Here we summarize current knowledge of how dysregulated RNA modifiers influence glucose, lipid, amino acid, and mitochondrial metabolism, and then, discuss the metabolic effects on RNA modifications.

### Glucose metabolism

**Fig. 3:**
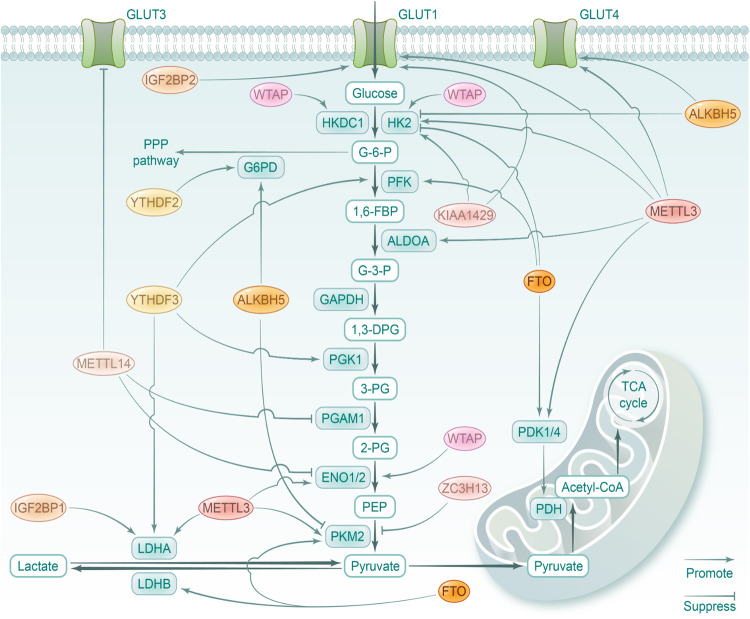
The roles of RNA modifications in glucose metabolism. The schematic diagram shows the direct regulation of RNA modification on glucose metabolism pathways. The key glycolytic enzymes, such as hexokinase (HK), enolase (ENO), Aldolase A (ALDOA), pyruvate kinase isozyme M1/2 (PKM1/2), pyruvate dehydrogenase kinase (PDK), lactate dehydrogenase (LDH) and glucose transporter (GLUT) are crucial targets of dysregulated RNA modifiers, and are generally upregulated in various pathologies. The figure is generated with BioRender (https://biorender.com)

Glucose is the main energy source of cells, the metabolic pathways principally include aerobic oxidation, anaerobic digestion, pentose phosphate pathway (PPP), glycogen synthesis and gluconeogenesis. Glycolysis is the fundamental energy-producing process in organisms, in which glucose is decomposed into pyruvate with free energy released into ATP.^191^ Normally, glycolysis in the cytosol is followed by mitochondrial oxidative phosphorylation (OXPHOS) to produce a large amount of ATP under aerobic condition. While in cancer cells, glycolysis had priority over mitochondrial respiration even with sufficient oxygen supply, known as Warburg effect or aerobic glycolysis.^192^ The key glycolytic enzymes, such as hexokinase (HK), enolase (ENO), Aldolase A (ALDOA), pyruvate kinase isozyme M1/2 (PKM1/2), pyruvate dehydrogenase kinase (PDK), lactate dehydrogenase (LDH) and glucose transporter (GLUT) are crucial targets of RNA modifications in various pathological processes. The pentose phosphate pathway (PPP) is next to glycolysis and the tricarboxylic acid (Krebs) cycle, subdivided into two branches, known as the oxidative and non-oxidative PPP. The non-oxidative PPP is virtually ubiquitous and can occur non-enzymatically, supporting biosynthesis of aromatic amino acid and RNA backbone with ribose 5-phosphate and erythrose 4-phosphate.^193^ The oxidative branch depends on glucose-6-phosphate (G-6-P) to produce ribulose-5-phosphate, carbon dioxide, and nicotinamide adenine dinucleotide phosphate (NADPH), absent in many aerobic and thermophilic organisms.^194^ Glycogen synthesis is catalyzed by glycogen synthase under balanced phosphorylation/de-phosphorylation of various kinases, exemplified by glycogen synthase kinase 3 (GSK-3). In fasting state, GSK-3 is activated through de-phosphorylation, thus inhibits glycogen synthesis and facilitates glycogenolysis. While normal feeding inactivated GSK-3 and promotes glycogen synthesis.^195^ Gluconeogenesis refers to the process that cells synthesize glucose or glycogen from non-sugar precursors such as lactic acid, glycerol, and amino acids. The liver gluconeogenesis is enhanced by decreased insulin and increased glucagon. Remarkably, RNA modifications have been confirmed to play crucial roles in glucose metabolic pathways via directly or indirectly regulating expression of glycolytic-related genes (Fig. 3 and Table 4).

**Table 4:** RNA modifications in glucose metabolism

Regulator	Disease	Target	Mechanism	Ref.
IGF2BP2	DM	PDX1	Stabilizes the transcripts in a m6A-dependent manner	^199^
		p53	Stabilizes the transcripts in a m6A-dependent manner	^200^
YTHDF2		PARP1	Reduce its expression	^196^
METTL14		LncRNA TINCR	Promote its degradation via YTHDF2	^201^
FTO		FOXO1/G6P/DGAT2	Upregulates the expression	^205^
METTL3	CC	PDK4	Promotes its translation elongation and mRNA stability of PDK4 via YTHDF1/eEF-2 complex and IGF2BP3	^210^
	LUAD	ENO1	Stabilizes the transcripts via YTHDF1	^212^
	CRC	HK2/GLUT1	Stabilizes the transcripts via IGF2BP2/3	^213,214^
	CRC	LDHA	Promotes its transcription via stabilizing HIF-1α mRNA, and triggers its translation via YTHDF1	^217^
	CRC	GLUT1/PKM2/LDHA/ALDOA	Indirect activation via IGF2BP2-mediated stabilization of PTTG3P mRNA	^218^
	ESCA	HK2/GLUT1	Promotes the expression	^215^
	PDAC	HK2	Upregulates its expression	^216^
	GC	ENO2/GLUT4	Indirect activation via IGF2BP3-mediated stabilization of HDGF mRNA	^219^
	NSCLC	MYC	Upregulates its expression via m6A/DLGAP1-AS2/YTHDF1 axis	^220^
	ESCA	MYC	Upregulates its expression via m6A/YTHDF/APC/β-catenin axis	^221^
	BRCA	YAP	Activates it via YTHDF2-mediated decay of LATS1	^223^
METTL14	CRC	SLC2A3/PGAM1	Decreases the expression through YTHDF2-mediated processing of pri-miR-6769b and pri-miR-499a	^231^
	HCC	SIRT6	Stabilizes USP48 to mediate deubiquination of SIRT6	^232^
	RCC	ENO2/SRC	Destabilizes BPTF to activate ENO2 and SRC	^233^
	GC	LHPP	Upregulates its expression	^234^
WTAP	GC	HK2	Stabilizes the transcripts	^235^
	OVC	HK2	Indirectly upregulates HK2 via interacting with DGCR8 to boost miR-200 maturation	^236^
	BRCA	ENO1	Facilitates the expression	^237^
	COAD	SMARCE1	Stabilizes FOXP3 via YTHHDF1 to transcriptionally activate SMARCE1	^238^
	CRC	HKDC1	Suppresses NT5DC3 expression to upregulate HKDC1	^239^
KIAA1429	CRC	HK2	Upregulates the expression	^240^
	GC	GLUT1	Upregulates LINC00958 to stabilize GLUT1 mRNA	^241^
RBM15	OS	HK2/GPI/PGK1	Upregulates the expression	^242^
ZC3H13	HCC	PKM2	Destabilizes its transcripts	^243^
FTO	HCC	PKM2	Promotes its expression	^244^
	GBM	PDK1	Promotes its expression	^245^
	AML	PFKP/LDHB	Upregulates the expression via YHTDF2	^247^
	CC	HK2	Partially reverses E6E7-induced improvement	^249^
	LUAD	MYC	Suppresses m6A/YTHDF1-mediated translation	^250^
	PTC	APOE	Attenuates m6A/IGF2BP2-dependent stabilization	^251^
ALKBH5	Glioma	G6PD	Stabilizes its transcripts	^252^
	BRCA	GLUT4	Protects GLUT4 from YTHDF2-mediated decay	^253^
	PTC	PKM2	Decreases circNRIP1 to suppress PKM2 expression	^254^
	HCC	HK2	Elevates UBR7 to suppress HK2 expression via Nrt/Bach1	^255^
YTHDF2	LC	G6PD	Promotes its translation	^257^
	CRC	G6PD	Reduces its ubiquitination via circ_0003215/miR-663b/DLG4 axis	^258^
	CRC	GSK3	Enhances its stability	^259^
	CRC	GSK3	Promotes degradation of STEAP3 to protect GSK3 from phosphorylation	^260^
YTHDF3	HCC	PFKL	Promotes its expression	^261^
	PDAC	LDHA/HK2/PGK1/SLC2A1	Mediates destabilization of lncRNA DICER1-AS1 to upregulate glycolytic genes	^262^
IGF2BP1	GC	MYC	Mediates stabilization role	^264^
	ccRCC	LDHA	Mediates stabilization role	^265^
IGF2BP2	CC	MYC	Stabilizes the transcripts	^266^
	OSCC	HK2	Stabilizes the transcripts	^267^
	HCC	HK2/GLUT1	miR4458HG interacts with IGF2BP2 to promote HK2 and GLUT1 expression	^268^
IGF2BP3	OSCC	GLUT1	Interacts with circFOXK2 to stabilize GLUT1 mRNA	^269^
	GC	MYC	LOC101929709 binds to LIN28B and IGF2BP3 to stabilize MYC mRNA	^270^
ALKBH3	HeLa cells	ATP5D	Upregulates it expression	^271^
ALYREF	BLCA	PKM2	Stabilizes the transcripts	^272^
METTL1	ACC	HK1	Promotes its expression	^273^
METTL3	HF	AR	Reduces its expression via YTHDF2	^275^
	Metabolic bone disease	ACLY/SLC25A1	Stabilizes the transcripts via IGF2BP2 and IGF2BP3	^276^

#### Diabetes mellitus

Type 2 diabetes (T2D) is characterized by insulin resistance and hyperglycemia. And functional integrity of β-cell in pancreatic islet is indispensable for glucose homeostasis. It has been demonstrated that high glucose concentrations reduce m6A level in human and mouse islets.^196^ Notably, m6A modification played a vital role in pancreatic beta-cell biology. In β-cell specific METTL14-knockout mice, dysfunction of islet, manifested as reduced β-cell proliferation and insulin degranulation, was observed, accelerating the occurrence of diabetes.^197^ Accordingly, Wang et al. revealed the essential role of METTL3/14 in beta-cell functional maturity. Depletion of METTL3/14 in endocrine progenitors implicated that METTL3/14 were dispensable for beta-cell differentiation but modulated expression of an essential transcription factor MAFA, leading to hypo-insulinemia and hyperglycemia.^198^ The m6A reader IGF2BP2 is identified as crucial for β-cell proliferation, PDX1 expression level, insulin secretion, and further related with T2DM susceptibility. Mechanistically, IGF2BP2 could stimulate PDX1 translation in an m6A dependent manner and orchestrate IGF2-AKT-GSK3beta-PDX1 signaling to stabilize PDX1 polypeptides.^199^ And IGF2BP2 is involved in restraining cardiac fibrosis in diabetic heart through LncRNA Airn /IGF2BP2/p53 axis in an m6A-dependent manner.^200^ Sun et al. figured out that YTHDF2-mediated m6A modification suppress the expression of poly (ADP-ribose) polymerase 1 (PARP1), which is indispensable in the progression of diabetic retinopathy (DR).^196^ Moreover, METTL14-mediated m6A mitigates diabetic cardiomyopathy via promoting the degradation of LncRNA TINCR dependent on YTHDF2.^201^ Particularly, FTO gene polymorphism rs9939609 and rs9940128 are closely associated with hyperglycemia, insulin resistance and diabetes mellitus in different populations.^202–204^ In T2D patients, FTO, METTL3, METTL14, and WTAP are upregulated and global m6A level was reduced. And FTO was positively correlated with serum glucose and expression level of several glucose-metabolic genes, such as forkhead box protein O1 (FOXO1), glucose-6-phosphate (G6P) and diacylglycerol O-acyltransferase 2 (DGAT2).^205^ Thereinto, FOXO1, as an essential transcription factor in gluconeogenesis, has been verified as a direct substrate of FTO. And the potential FTO inhibitor entacapone elicits glucose-lowering function in vivo.^206^ Moreover, unregulated activating transcription factor 4 (ATF4) was found in FTO-overexpressed transgenic mice, which could augment glucose production by modulating G6P.^207,208^ Moreover, m6A modification exerts regulatory roles in insulin resistance (IR). Hu et al. proposed that inhibition of FTO aggravates the insulin resistance and adipose tissue inflammation in T2D mice.^209^

#### Cancer

Abnormal glucose metabolism, manifested as enhanced glycolytic activity and lactic acid fermentation, is a fundamental part of tumor metabolic reprogramming. Numerous studies have revealed that METTL3-induced m6A directly upregulated expression of various glycolytic enzymes in different cancers. In cervical cancer (CC) cells, METTL3 promotes the translation elongation and mRNA stability of PDK4 depending on YTHDF1/eEF-2 complex and IGF2BP3, respectively.^210,211^ In lung adenocarcinoma (LUAD), METTL3/m6A/YTHDF1 augment the stability of ENO1 mRNA.^212^ In colorectal cancer (CRC), METTL3 catalyzes m6A on 5’/3’UTR of HK2 and 3’UTR of GLUT1 (SLC2A1), further stabilizing the transcripts through IGF2BP2 or IGF2BP2/3, respectively.^213^ Consistently, Chen et al. identified that METTL3/m6A/GLUT1/mTORC1 axis, and overexpression of METTL3 could predict poor survival of CRC patients.^214^ In esophageal carcinoma (ESCA), the multivariate analysis confirmed the positive association between METTL3 level and expression of GLUT1 and HK2.^215^ And Li et al. have verified its enhancement on HK2 expression in PDAC cells.^216^ In 5-FU resistant CRC cells, overexpressed METTL3 not only promoted the transcription of LDHA via stabilizing mRNA of HIF-1α, but also triggered its translation in a YTHDF1-dependent manner.^217^ And METTTL3 could indirectly activate expression of GLUT1, ALDOA, PKM2, and LDHA in CRC cells via IGF2BP2-mediated stabilization of PTTG3P mRNA.^218^ Similar indirect activation on GLUT4 and ENO2 was achieved via IGF2BP3-mediated stabilization of HDGF mRNA in GC cells.^219^

Aside from above glycolytic-related key enzymes or transporters, METTL3 exerts extensive regulation on other metabolic-related targets to motivate glycolysis. Known as a wide-ranging oncogenic determinant, c-MYC was found to be upregulated by METTL3 via m6A/DLGAP1-AS2/YTHDF1 in non-small cell lung cancer (NSCLC)^220^ and m6A/YTHDF/APC/β-catenin in ESCA, further advancing glycolytic metabolism.^221^ HIF-1α is responsible for hypoxia conditions in tumor environment, which form mutual feedback with tumor growth. METTL3-induced m6A modification positively regulates HIF-1α level, leading to enhanced aerobic glycolysis.^222^ METTL3 could also regulate glycolysis and tumorigenesis of breast cancer (BRCA) via YAP, the downstream of Hippo pathway. In mechanism, YTHDF2 accelerated degradation of m6A-modified LATS1 mRNA, thus reduced phosphorylation of YAP/TAZ and activated it.^223^ In addition to these compelling transcription factors (TFs), METTL3 boosted expression of NDUFA4 in GC,^224^ AKR1B10 in cholangiocarcinoma (CCA),^225^ NCAPH in clear cell renal cell carcinomas (ccRCC),^226^ thus promoted glycolysis and malignant phenotypes. Notably, METTL3-induced m6A interacted with ncRNAs to improve glycolysis, such as stabilizing effects on lncRNA ABHD11-AS1 in NSCLC,^227^ lncRNA SNHG7 in prostate cancer (PC),^228^ circQSOX1 in CRC,^229^ and linc-UROD in PC,^230^ which are generally mediated by IGF2BPs.

Interestingly, METTL14 seems to exert negative influences on tumor glucose metabolism. In CRC, METTL14 repressed glycolysis via YTHDF2-dependent processing of pri-miR-6769b and pri-miR-499a, which attenuated SLC2A3 and PGAM1 expression, respectively.^231^ In HCC, METTL14 stabilizes USP48 mRNA, which mediated deubiquitination at the K33 and K128 sites of SIRT6, thus hindered glycolytic reprogramming.^232^ In RCC, METTL14 attenuated stability of BPTF mRNA, which constituted super-enhancers that activated downstream glycolysis-related genes like ENO2 and SRC.^233^ Lin et al. proposed that METTL14 positively regulated LHPP expression to restrain aerobic glycolysis of GC.^234^

WTAP, another m6A writer, was identified to promote Warburg effect in several cancers. WTAP targets 3′-UTR of HK2 mRNA and increased its stability in GC,^235^ while it indirectly upregulates HK2 via interacting with DGCR8 to boost miR-200 maturation in Ovarian Cancer (OVC).^236^ Ou et al. supplemented that WTAP-induced m6A methylation could facilitate expression of ENO1 in BRCA.^237^ In Colon adenocarcinoma (COAD), WTAP stabilizes FOXP3 mRNA via YTHDF1, and FOXP3 bound to SMARCE1 promoter to exert transcriptional activation.^238^ In CRC, WTAP modifies NT5DC3 to suppress the tumorigenesis under hyperglycemia via repressing Hexokinase domain component 1 (HKDC1).^239^ Besides, writer KIAA1429 upregulates HK2 and GLUT1 level in methyltransferase activity-dependent manner, facilitating glycolytic process of CRC and GC,^240,241^ and RBM15 catalyzes m6A modification to accelerate expression of HK2, glucose-6-phosphate isomerase (GPI) and phosphoglycerate kinase1 (PGK1) in OS,^242^ while ZC3H13 significantly destabilizes PKM2 mRNA to weaken glycolytic reprogramming and enhance cisplatin sensitivity of HCC.^243^

FTO, the m6A eraser, demonstrates an ambiguous role in regulating glycolytic metabolism. FTO-triggered demethylation was found to enhance glycolysis of HCC and GBM via directly facilitating expression of key enzymes PKM2 and PDK1.^244,245^ Especially, studies have confirmed the suppressive effect on glycolysis of some selective FTO inhibitors. In SCLC cell line, meclofenamic acid (MA) treatment significantly induced attenuated glycolysis and enhanced mitochondrial metabolism.^246^ R-2-hydroxyglutarate (R-2HG) represses aerobic glycolysis of leukemia cells via abrogating m6A/YTHDF2-mediated upregulation of PFKP and LDHB, thus inhibiting leukemogenesis in vivo.^247^ Besides, FTO elevates TFs c-Jun, JunB, and C/EBPβ to upregulate glycolysis-related genes in melanoma, contributing to escaping immune surveillance. Targeting FTO with a small compound Dac51 successfully stimulated therapeutic benefit of anti-PD-L1 blockade.^248^ Nevertheless, several researchers proposed opposite conclusions. Recently, Liu et al. reported that overexpression of FTO could partially reverse E6E7-induced improvement on HK2 in CC.^249^ In LUAD, Wnt signaling induces downregulation of FTO, thus increased m6A level leads to enhanced YTHDF1-mediated translation of c-MYC and subsequently increases glycolysis.^250^ FTO diminishes IGF2BP2-dependent stabilization of APOE mRNA, thus restrains glycolysis and growth of papillary thyroid cancer (PTC).^251^

Likewise, another m6A eraser, ALKBH5 also have dual regulatory effects. ALKBH5 enhanced stability of G6PD mRNA, thereby activating PPP and promoting proliferation of glioma cells.^252^ In HER2 resistant BRCA cells, ALKBH5 stimulated glycolysis via protecting GLUT4 mRNA from YTHDF2-mediated decay.^253^ However, in PTC, knockdown of ALKBH5 accelerates glycolysis through upregulating circNRIP1 and consequently increased PKM2 expression.^254^ Zhao et al. discovered that overexpressed ALKBH5 elevated expression level of UBR7, which inhibited glycolysis by indirectly suppressing HK2 expression through Nrf2/Bach1 axis.^255^

Particular attention has been given to m6A readers, which recognize m6A marks and mediate highly context-specific regulation on glycolytic process. Generally, YTHDF1 positively controls glycolysis through stabilizing transcripts or initiating translation in a wide range of cancers.^210,212,256^ Consistent to its binary regulation on gene expression, YTHDF2 indeed plays diverse roles in reprogramming glycolytic metabolism, with underlying rationale to be further elucidated. For instance, YTHDF2 accelerates decay of GLUT4 mRNA in BRCA,^253^ while facilitates expression of PFKP and LDHB in leukemia,^247^ leading to opposite effects. Meanwhile, YTHDF2 participates in modulating other glucose metabolic pathways of glucose like PPP and glycogen synthesis. In LC, overexpressed YTHDF2 binds to m6A sites on 3′-UTR of G6PD mRNA to promotes its translation, enhancing PPP flux,^257^ and Chen et al. proposed that YTHDF2 enhanced PPP via reducing G6PD ubiquitination by circ_0003215/miR-663b/DLG4 axis.^258^ In CRC, YTHDF2 is capable to stabilize mRNA of GSK3 to inhibit glycogen synthesis and facilitate glycogenolysis.^259^ Also, YTHDF2-mediated degradation of STEAP3 mRNA attenuated STEAP3-induced phosphorylation and inactivation of GSK3β in CRC.^260^

YTHDF3 facilitated aerobic glycolysis of HCC cells by elevating PFKL expression, and PFKL in turn upregulated YTHDF3 through reducing its ubiquitination.^261^ In PDAC, YTHDF3-mediated destabilization of lncRNA DICER1-AS1 contributes to enhancing expression of glycolytic genes like LDHA, HK2, PGK1, and SLC2A1,^262^ while YTHDF3 targeted m6A-modified PGK1 mRNA to exert a stabilizing role in OS.^263^ IGF2BP1 was highly expressed in GC tissue and associated with poor prognosis for GC patients. IGF2BP1 promoted the migration and aerobic glycolysis of GC cells via directly interacting with c-MYC mRNA to stabilize it.^264^ The gain/loss functional assays proved IGF2BP1-mediated stabilization of LDHA mRNA in ccRCC.^265^ Moreover, upregulated IGF2BP2 has been found as a predictor of poor prognosis in CC and OSCC, which improved stability of c-MYC and HK2 mRNA, respectively.^266,267^ Recently, Ye et al. suggested that miR4458HG interacted with IGF2BP2 and activated the improvement of HK2 and GLUT1 expression in HCC.^268^ In OSCC, IGF2BP3 interacted with circFOXK2 to stabilize GLUT1 mRNA.^269^ And LOC101929709 bound to LIN28B and IGF2BP3, facilitating LIN28B to stabilize m6A-modified c-MYC mRNA in GC.^270^

Recently, Wu et al. demonstrated the interplay between m1A modification and tumor glycolytic metabolism. In HeLa cells, ALKBH3 promoted glycolysis by upregulating ATP5D, a subunit of mitochondrial ATP synthase. Mechanistically, the m1A marks on ATP5D mRNA hinders its translation elongation via recruiting YTHDF1/eRF1 complex, and m1A modification destabilizes E2F1 mRNA to block the initiation of ATP5D transcription. ALKBH3^-/-^ HeLa cells displayed reduced glycolysis and weakened growth, both depletion m1A of ATP5D by dm1ACRISPR and overexpression of ATP5D could recede the suppression effect.^271^ In bladder cancer (BLCA), m5C reader ALYREF bound to 3′-UTR of PKM2 mRNA to stabilize it, and HIF-1α exerted indirect activation on ALYREF in this process.^272^ Bioinformatics studies have preliminarily implicated that m7G modification participated in glycolytic metabolism. In adrenocortical carcinoma (ACC), a novel m7G risk signature consisted of METTL1, NCBP1, NUDT1 and NUDT5 was constructed, and the risk score presented significant correlation with enrichment of glycolysis. Especially, METTL1, was found to positively regulate the expression of HK1.^273^

#### Other diseases

Epigenetic influence of RNA modification on dysregulated glycolysis has been noted in several other pathological processes. Zhang et al. first investigated the role of FTO as a m6A eraser in cardiac metabolism and suggested that FTO could attenuate cardiac dysfunction by regulating glucose uptake and glycolysis with pressure overload-induced heart failure (HF) in mice. Future studies are warranted to systematically assess the potential of FTO for HF prevention and treatment.^274^ For cardiac fibrosis, METTL3 could repress androgen receptor (AR) expression in a YTHDF2-dependent manner, which activates HIF-1α signaling, thus enhancing glycolysis and cardiac fibroblast proliferation.^275^ Cai et al. revealed the potential metabolic-related regulation of RNA modification in osteogenic differentiation, inspiring future clinical applications in metabolic bone diseases and stem cell therapy. The mechanistic study showed that METTL3 enhanced stability of ATP citrate lyase (ACLY) and a mitochondrial citrate transporter (SLC25A1) mRNA mediated by IGF2BP2 and IGF2BP2/3, respectively.^276^ In palmitate (PA)-induced IR C2C12 cells and high-fat diet (HFD)-fed mice model, Quercetin downregulated METTL3, lead to decreased phosphorylated insulin receptor substrate 1 (p-IRS1) levels, increased serine-threonine kinase protein kinase D2 (PRKD2), GLUT4 and p-AKT, further enhancing glucose uptake and alleviating oxidative stress.^277^

### Lipid metabolism

**Fig. 4:**
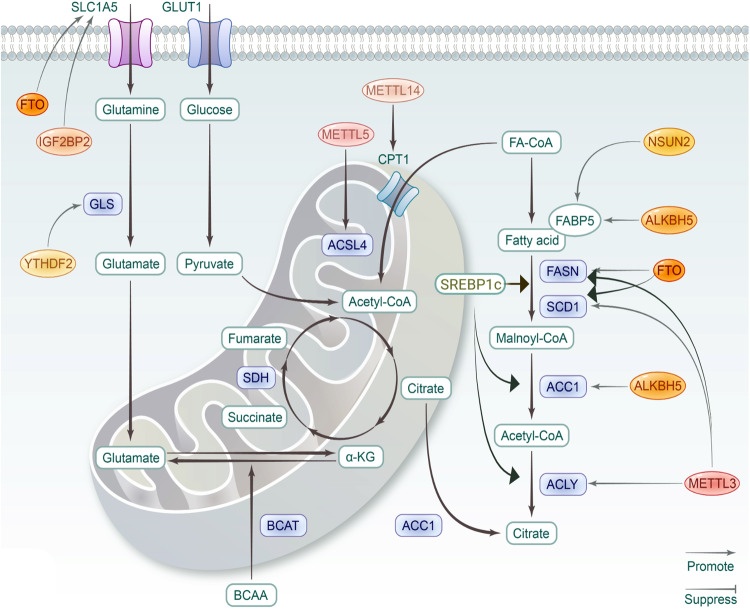
The roles of RNA modifications in lipid and amino acid metabolism. For lipid metabolism, key enzymes in FA synthesis, including fatty acid synthase (FASN), acetyl-CoA carboxylase (ACC), stearoyl-CoA desaturase1 (SCD1), and ACLY, are significant targets of RNA modifications. Relevant studies on amino acid metabolism are limited. The figure is generated with BioRender (https://biorender.com)

Lipids are essential components of biological membranes, building blocks of biosynthesis, and significant energy storage. According to the comprehensive classification system, lipids are categorized into fatty acyls (FA), glycerolipids (GL), glycerophospholipids (GP), sphingolipids (SP), sterol lipids (ST), prenol lipids (PR), saccharolipids (SL), polyketides (PK).^278^ FA could be esterified and stored in lipid droplets during high nutrient availability, while hydrolyzed to generate ATP by FA oxidation (FAO), also called β-oxidation, under energy stress conditions. FA synthesis is under control of sterol regulatory element-binding protein 1c (SREBP1c). Stimulated by growth factors, the precursor is processed into mature SREBP1c, and then translocated into nucleus to improve the transcription of target genes, including fatty acid synthase (FASN), acetyl-CoA carboxylase (ACC), stearoyl-CoA desaturase1 (SCD1), and ACLY.^279,280^ Cholesterol is the material for synthesis of fat-soluble vitamins and steroid hormones, and also the constitution of membranes, together with GL, GP and SP.^281^ Dysregulated lipid metabolism is implicated in several pathologies, with RNA modifications participating in various metabolic links (Fig. 4 and Table 5).

**Table 5:** RNA modifications in lipid, amino acid and mitochondrial metabolism

Regulator	Disease	Target	Mechanism	Ref.
Lipid metabolism				
METTL3	Obesity	FASN	Upregulates its mRNA level	^284^
		CCND1	Promotes degradation of CCND1 mRNA via YTHDF2	^285^
FTO		RUNX1T1	Controls the exonic splicing by regulating the RNA binding ability of SRSF2	^288^
		CCNA2/CDK2	Reduces the YTHDF2-mediated decay of mRNA	^289,290^
YTHDF1		MTCH2	Facilitate its translation	^296^
METTL3/14	NAFLD	ACLY/SCD1	Upregulates the expression	^299^
METTL3		Rubicon	Promotes Rubicon expression via YTHDF1	^300^
FTO		SREBP1/SCD1	Upregulates the expression via m6A demethylation	^303^
		IL-17RA	Upregulates its expression via m6A demethylation	^307^
ALKBH5		LINC01468	Stabilizes LINC01468 to promote degradation of INPPL1	^308^
		PTCH1	promotes its expression via m6A demethylation	^309^
YTHDF3		PRDX3	Facilitates its translation	^310^
METTL14	AS	p65	Promotes the expression of p65	^314^
		lncRNA ZFAS1	Downregulates ZFAS1 level	^315^
FTO		PPARγ	Downregulates expression level of PPARγ and promotes phosphorylation of AMPK	^317^
METTL14	BLCA	PPARs	METTL14 elevates level of lncDBETm, which interacts with FABP5 to activate PPARs	^321^
-	BRCA/HCC	CPT1B	Elevated m6A level triggers the splicing of precursor ESRRG mRNA to improve ERRγ, which upregulate CPT1B	^323^
METTL5	HCC	ACSL4	Promotes ACSL4-mediated FAO	^324^
FTO	HCC	FASN	Protects the mRNA from YTHDF2-mediated decay	^325^
FTO	EC	HSD17B11	Enhances its translation	^326^
ALKBH5	CESC	ACC1	Attenuates IGF2BP1-mediated stabilization of SIRT3, further reduces ACC1 level by repressing its deacetylation	^327^
YTHDF2	GBM	LXRA/HIVEP2	Facilitates decay of mRNAs to suppress cholesterol synthesis, efflux, and uptake	^329^
	CRC	DEGS2	Mediates its degradation to induce lipidomic dysregulation	^330^
IGF2BP2	AML	MFSD2A	Stabilizes PRMT6 mRNA to suppress MFSD2A expression	^331^
HNRNPA2B1	GC	RPRD1B	Stabilizes the transcripts	.^332^
	ESCA	ACLY/ACC1	Promotes the expression	^333^
NSUN2	OS	FABP5	Stabilizes the transcripts	^335^
TRMT6/TRMT61A	HCC	PPARδ	Facilitates its translation	^337^
Mitochondrial metaboliam				
FTO	ccRCC	PGC-1α	Upregulates its expression	^341^
METTL3	NSCLC	DCP2	Accelerates its degradation	^342^
	BRCA	AK4	Upregulates AK4 to ROS production and p38 phosphorylation	^343^
METTL14	CRC	miR-17-5p	Induces degradation of miR-17-5p via YTHDC2, which downstream suppress MFN2	^345^
IGF2BP1	GC	NDUFA4	Upregulates NDUFA via stabilization to enhance oxidative metabolism	^224^
IGF2BP2	GBM	SHMT2	Stabilizes its mRNA to promote OXPHOS	^350^
RALY	CRC	ETC-related genes	Augments processing of pri-miRNA to further downregulate ETC-related genes	^352^
Amino acid metabolism				
FTO	ccRCC	SLC1A5	Promotes its expression	^357^
	CRC	ATF4	Upregulates ATF4 to activate DDIT4, and then suppress mTOR signaling	^358^
YTHDF1	CRC	GLS	induces translational promotion of GLS	^360^
IGF2BP2	AML	GPT2/SLC1A5/MYC	Enhances mRNA stability and translation of several glutamine metabolism-related genes	^361^
METTL16	AML	BCAT1/2	Facilitates its expression to regulate branched-chain amino acid metabolism	^363^

#### Obesity

In current cognition, obesity is the result of genetic and environmental factors, thereinto, epigenetic regulation such as RNA modifications play significant roles.

Transcriptome profile of human adipose tissue displayed that several m6A modifiers, including WTAP, VIRMA, ALKBH5, and YTHDC1, are associated with obesity and clinical variables, while single nucleotide polymorphisms of METTL3 correlates with body mass index (BMI).^282^ In brown adipose tissue (BAT), METTL3 is essential for the postnatal maturation and BAT-specific depletion of METTL3 accelerated development of HFD-induced obesity.^283^ Hepatocyte-specific ablation of METTL3 could promote fatty acid metabolism in mice fed with HFD through regulating fatty acid synthase (FASN), enhancing insulin sensitivity.^284^ METTL3/m6A/YTHDF2 mediate decay of cyclin D1 (CCND1) mRNA to block cell-cycle progression and inhibit adipogenesis.^285^

Since FTO was initially discovered as an obesity-related protein before as an eraser, its correlation with obesity has been widely reported in different populations.^33,286,287^ Significantly, FTO plays a critical role in lipogenesis and obesity susceptibility dependent on m6A demethylase activity. FTO could adjust exonic splicing of adipogenic regulatory factor runt-related transcription factor 1 (RUNX1T1) through eliciting m6A modifications around splice sites, further induces the differentiation of mouse 3T3-L1 preadipocytes.^288^ FTO could restrain cell cycle progression of preadipocytes and adipogenesis via YTHDF2-dependent decay of cyclin A2 (CCNA2) and cyclin dependent kinase 2 (CDK2).^289,290^ Also, Zinc finger protein (ZFP217) regulate adipogenesis via FTO/m6A/YTHDF2 axis.^291^

FTO-mediated demethylation facilitates the expression of peroxisome proliferator-activated receptor gamma (PPARG) mRNA, which promotes the differentiation of bone marrow stem cells (BMSCs) into adipocytes.^292^ Although depletion of endothelial FTO has no effect on the development of obesity and dyslipidemia, it could promote AKT (protein kinase B) phosphorylation in endothelial cells and skeletal muscle to preserve myogenic tone in resistance arteries, which ultimately alleviates obesity-induced hypertension.^293^ In accordance, AMP-activated protein kinase (AMPK) was found to regulate lipid metabolism of skeletal muscle via FTO-dependent m6A demethylation.^294^ Moreover, betaine-mediated downregulation of FTO contributes to dysfunctional adipose tissue induced by high-fat diet.^295^ YTHDF1 was identified to enhance translation of mitochondrial carrier homology 2 (MTCH2) mRNA and in an m6A-dependent way, which promoting lipogenesis.^296^ And YTHDF2 facilitated degradation of cyclin D1 mRNA to mediate adipogenesis inhibition.^297^

#### NAFLD

Metabolic disorders, manifested as dysregulated de novo lipogenesis, fatty acid uptake, fatty acid oxidation, and triglycerides export, are essential part of pathological mechanism of NAFLD.^298^ In NAFLD mice model, targeting METTL3/14 upregulated level of ACLY and SCD1, promoting cholesterol production and lipid droplet deposition.^299^ METTL3/m6A/YTHDF1 exerts a stabilizing effect of Rubicon mRNA and promotes its expression, leading to hepatic lipid deposition.^300^ m6A hypomethylation state and increased FTO level are detected in HFD-induced NAFLD mice, and FTO participates in the hepatoprotective effects of betaine.^301^ Overexpressed FTO significantly enhanced lipogenesis and oxidative stress in vitro.^302^ In the glucocorticoid (GC)-induced NAFLD model, glucocorticoid receptor (GR)-mediated FTO transactivation promotes hepatocyte adipogenesis and lipid accumulation via m6A demethylation of SREBF1 and SCD1.^303^ Exposure of endocrine disrupting chemicals (EDCs) was confirmed to induce NAFLD, in which process decreased global m6A level and altered expression of m6A modulators was observed.^304^ Inhibition of FTO effectively alleviates progression of dexamethasone-induced fatty liver in mice.^305^ Reduced FTO expression mediates the ameliorating effect of exenatide therapy on lipid accumulation and inflammatory responses in NAFLD.^306^ Moreover, FTO-mediated m6A demethylation increases interleukin-17 receptor A (IL-17RA) level in tumor adjacent tissues with chronic inflammation, suggesting the potential role of FTO in inflammation-carcinogenesis transformation of HCC.^307^

And ALKBH5-dependent demethylation drives lipogenesis and NAFLD-HCC progression via stabilizing LINC01468, which accelerates cullin4A (CUL4A)-linked degradation of inositol polyphosphate phosphatase-like 1 (INPPL1, SHIP2).^308^ Nevertheless, another study demonstrated that overexpressed ALKBH5 could ameliorate liver fibrosis and inactivate Hepatic stellate cells (HSCs) via upregulating Patched 1 (PTCH1).^309^ Moreover, YTHDF3 was also reported to restrain liver fibrosis and HSC activation via facilitating peroxiredoxin 3 (PRDX3) translation in an m6A-dependent manner.^310^

#### Atherosclerosis

During the development and progression of atherosclerosis (AS), RNA modifications play critical roles in lipid deposition and fiber cap formation.^311^ Growing evidences showed that RNA modifications participate in development of AS via modulating inflammatory cell infiltration and immune response, including vascular endothelial cells, macrophages, and vascular smooth muscle cells (VSMCs).^312^

In an ox-LDL-induced AS model, highly expressed METTL3 in VSMCs facilitates the binding of DGCR8 to pri-miR-375 and further improved miR-375-3p expression, which targets PDK1 transcription, inducing phenotypic transformation of VSMCs and rendering AS plaques more vulnerable.^313^ In ox-LDL-treated human umbilical vein endothelial cells (HUVEC), upregulated METTL3 and METTL14 were detected, and METTL14 modified p65 mRNA to facilitate lipoprotein synthesis and AS development.^314^ METTL14 was found to reduce cholesterol efflux and enhanced atherosclerotic plaque inflammation via modifying lncRNA ZFAS1.^315^ FTO exerts various effects on vascular homeostasis properties via influencing lipid metabolism. Researchers have reported the anti-atherosclerotic properties of FTO. In-vivo experiments showed that overexpressed FTO induced by Adeno-associated virus serotype 9 (AAV9) obviously decreased the lipidic profiles including plasma total cholesterol and LDL cholesterol, and mitigated the formation of atherosclerotic plaques.^316^ Yang et al. demonstrated that FTO inhibits macrophage lipid influx by downregulating PPARγ expression and facilitating cholesterol efflux via phosphorylation of AMPK, thereby meliorating foam cell formation and AS development.^317^ Also, Kruger et al. found that endothelial-specific knockdown of FTO could prevent obesity-induced vascular dysfunction.^293^

#### Cancer

Activated de novo synthesis of fatty acids serves as an essential energy source, while enhanced FAO contributes to ATP production, intracellular ROS reduction. Except for bioenergetic demand, remodeled lipid metabolism could assist tumor development through modulating ferroptosis, enabling metastasis and invasion, and crosstalk with other hallmarks in TME.^318–320^

In BLCA, METTL14-mediated m6A elevates level of lncDBETm, which interacts with Fatty acid-binding protein 5 (FABP5) to activate peroxisome proliferator-activated receptors (PPARs), markers of lipid metabolism-related signaling pathways.^321^ And previous study suggested that METTL3 could recruit YTHDF2 to stabilize PPARα mRNA, regulating circadian rhythms of hepatic lipid metabolism.^322^ In breast and liver cancer cell, elevated m6A modification upregulated ERRγ by triggering the splicing of precursor ESRRG mRNA, subsequently improved expression of carnitine palmitoyl transferase 1B (CPT1B), a rate-limiting enzyme of FAO, conferring to chemoresistance.^323^ Peng et al. confirmed that METTL5 promoted de novo lipogenesis and HCC progression via ACSL4-mediated FAO. Targeting ACSL4 and METTL5 cooperatively suppresses HCC tumorigenesis in vivo.^324^

Overexpressed FTO enhances lipogenesis and lipid droplet enlargement in liver, and inhibits CPT1-mediated FAO via the SREBP1c pathway. FTO-dependent m6A demethylation indirectly elevates SREBP1c expression, thus activating downstream lipogenic genes.^303^ Knockdown of FTO markedly enhanced m6A abundance of FASN mRNA and promoted YTHDF2-mediated decay, further reduced protein levels of ACC1 and ACLY, which suppressed de novo lipogenesis in HepG2 cells.^325^ In EC, mechanism study revealed that FTO promoted the formation of lipid droplets by enhancing HSD17B11 expression.^326^ ALKBH5 was downregulated in cervical squamous cell carcinoma (CESC) and predicted an unfavorable prognosis. ALKBH5 attenuated stability of SIRT3 mRNA in an IGF2BP1-dependent manner, further reduced ACC1 level repress its deacetylation, thus suppresses fatty acid synthesis to modulate CESC lipid metabolism.^327^

YTHDF2 was reported to targets m6A-marked transcripts of key lipogenic genes to induce their degradation, thus suppressing liver steatosis.^328^ In GBM, YTHDF2 facilitates decay of LXRA and HIVEP2 mRNA, negatively regulating cholesterol synthesis, efflux, and uptake.^329^ In CRC, reduced m6A methylation promoted DEGS2 expression via attenuating YTHDF2-mediated decay, which contributed to dysregulated lipid metabolism, especially suppressed ceramide synthesis.^330^ In AML, IGF2BP2 stabilizes PRMT6 mRNA through m6A-dependemt manner, which catalyzes H3R2me2a and suppresses lipid transporter MFSD2A expression, thus decreasing docosahexaenoic acid levels and promoting LSC maintenance.^331^ HNRNPA2B1 participates in enhancing fatty acid metabolism of GC via stabilizing RPRD1B mRNA, which facilitated uptake and synthesis of FA by transcriptionally activating c-Jun/c-Fos, further upregulated SREBP1.^332^ Abrogation of HNRNPA2B1 inhibits de novo fatty acid synthesis in ESCA cells though downregulating expression of ACLY and ACC1.^333^

Several studies have dictated the regulatory functions of m5C modification in lipid metabolism. Function analysis demonstrated that highly m5C-marked genes were enriched in pathways correlated with decreased adipogenesis and improved myogenesis. Particularly, reader ALYREF recognized m5C targets on YBX2 and SMO and mediated the shuttling from nucleus to cytoplasm, thereby regulating adipogenesis and myogenesis, implicating a novel therapeutic approach for metabolic disorder diseases.^334^ The m5C writer NSUN2 has been found to advance adipogenesis through targeting CDKN1A mRNA and recruiting ALYREF to facilitate its nuclear export, thus accelerating cell cycle progression to promote lipid accumulation in preadipocytes.^133^ In OS, NSUN2-induced m5C modification stabilized FABP5 mRNA to positively regulated FA metabolism, further enhancing OS progression.^335^ In silico analysis identified that two clusters OVC samples with different m5C modification pattern exhibited distinct metabolic characteristics, with distinct expression profile of lipid metabolism-related pathways.^336^ In HCC, the m1A methyltransferase complex, TRMT6/TRMT61A facilitate PPARδ translation, which augmented cholesterol synthesis to initiate Hedgehog signaling, eventually driving self-renewal of liver CSCs and tumorigenesis.^337^

### Mitochondrial metabolism

Mitochondrion, the metabolic center in cells, plays an indispensable role in oncogenesis. Although aerobic glycolysis occupies an essential position in tumor bioenergetic metabolism, the OXPHOS and mitochondria-dependent energy supply were considered as key to maintain the stemness of some tumor cells.^338^ Besides, providing materials for anabolism, producing ROS, and maintaining regulated cell death (RCD) signaling significantly conduce to tumor progression.^339^

Peroxisome proliferator-activated receptor gamma coactivator 1-alpha (PGC-1α) is an essential cofactor for mitochondrial biogenesis and energy metabolism, which play significant parts in pathologies of monocyte-macrophage inflammation-associated diseases, such as atherosclerosis and rheumatoid arthritis. METTL3/m6A/YTHDF2 mediated degradation of PGC-1α, as well as cytochrome c (CYCS) and NADH ubiquinone oxidoreductase subunit C2 (NDUFC2), inducing mitochondrial dysfunction and oxLDL-induced inflammation in monocytes.^340^ During myoblasts differentiation, FTO positively modulates mTOR-PGC-1α pathway-mediated mitochondria biogenesis depending on its m6A demethylase activity.^32^ Consistently, ectopic expression of FTO in VHL-deficient ccRCC cells upregulated expression of PGC-1α to regain mitochondrial activity, exhibiting anti-proliferation effect.^341^

In NSCLC, METTL3-induced m6A methylation on DCP2 mRNA accelerates its degradation, which activates mitochondrial autophagy through the Pink1-Parkin pathway, inducing resistance to cisplatin and etoposide.^342^ In amoxifen-resistant BRCA cells, METTL3 was overexpressed and contributed to upregulation of AK4, which stimulating ROS production and p38 phosphorylation, further suppressing mitochondrial apoptosis to desensitize tamoxifen. Additionally, METTL3 knockdown abrogated AK4 expression and drug resistance.^343^ Also, METTL3 exerts inhibitive role in mitochondrial apoptosis via AKT signaling pathway in EC.^344^ METTL14 plays a pivotal part in regulating mitochondrial homeostasis in CRC via m6A/YTHDC2/miR-17-5p/MFN2 axis. Low expression of METTL14 ultimately led to less apoptosis and 5-FU chemoresistance in CRC.^345^ METTL14-catalyzed m6A increases expression of Fission 1 (FIS1), contributing to cadmium (Cd)-induced mitochondrial fission and dysfunction.^346^

FTO-mediated demethylation exerts protective roles in progression of hepatic ischemia-reperfusion injury (HIRI) via targeting dynamin-related protein 1 (Drp1) to alleviate liver oxidative stress and mitochondrial fragmentation in vivo and in vitro.^347^ Moreover, in silico analysis showed that overexpressed FTO and METTL5 were significantly associated with OXPHOS in NSCLC and GC, respectively.^348,349^ IGF2BP1 augmented stability of m6A-modified NDUFA4, and upregulated NDUFA4 enhanced oxidative metabolism in GC cells, whereas suppression of mitochondrial fission could switch the NDUFA4-induced mitochondrial activities and tumor growth of GC.^224^ In GBM, IGF2BP2 was involved in maintaining stability of SHMT2 mRNA, which played a crucial role in OXPHOS activities, as a significant driving force of GBM tumorigenesis.^350^ Elevated m6A level and IGF2BP2 expression conduced to maintenance of hematopoietic stem cells (HSCs) via restricting mitochondrial activity. IGF2BP2 deficiency accelerated degradation of Bmi1 mRNA, relieving its depression on mitochondria-related genes, thus impaired quiescence state and functions of HSCs.^351^ A novel RNA-binding protein, RALY, a member of HNRNPC subfamily, augmented pri-miRNA processing via the METTL3-mediated m6A modification, further reprogrammed mitochondrial metabolism by downregulating the electron transport chain (ETC) genes in CRC cells. Depletion of RALY demonstrated effective inhibition of tumor growth and development in vivo and in organoid models.^352^ In glioma, YTHDF1 participated in c-MYC-induced restraint on mitochondrial autophagy by directly interacting with FDX1 and upregulated its expression, which was closely correlated with malignant phenotypes and clinical prognosis.^353^ Highly-expressed HNRNPA2B1 served as an adverse prognostic factor in MM. HNRNPA2B1 recognized m6A sites at TLR4 and elevated its expression in post-transcriptional level, which protecting mitochondria under proteasome inhibitor exposure.^354^

### Amino acid metabolism

Except for serving as substrates for synthesizing proteins or peptides, amino acids are decomposed through deamination or transamination to generate building blocks for anabolism, like α-ketoacids utilized to release energy in TCA cycle. Particularly, glutamine has multiple biofunctions beyond a metabolic fuel or protein precursor. Glutamine decomposition is another significant feature of tumor metabolism remodeling.^355^ Given that cancers are normally auxotrophic for some non-essential amino acids, targeting the supply of these amino acids have been validated as an effective therapeutic intervention.^356^

Previous studies have substantiated the regulatory effects of m6A modification in glutamine metabolism. FTO-mediated m6A demethylation upregulated expression of the glutamine transporter SLC1A5, and FTO inhibition exclusively suppressed the proliferation and vitality of VHL-deficient ccRCC cells independent of HIF.^357^ Han et al. suggested that m6A modification exerted a significant role in the antitumor efficacy of glutaminolysis inhibition in CRC cells. FTO was upregulated upon glutaminolysis inhibition to promote expression of ATF4 via abrogating m6A/YTHDF2-mediated RNA degradation, and ATF4 activated pro-survival autophagy via transcriptionally upregulating DDIT4 to suppress mTOR signaling.^358^ Actually, it was found earlier that m6A modifications in the 5′ UTR of ATF4 mRNA modulated its alternative translation to mediate re-initiation independent of eIF2α signaling pathway, in response to amino acid starvation, implicating the translation regulation of m6A in integrated stress response.^359^ In CRC, YTHDF1 bound to 3′ UTR of glutaminase (GLS) mRNA and induced translational promotion, leading to enhanced glutamine uptake, further mediating chemoresistance, and targeting YTHDF1 effectively re-sensitizes CRC cells to cisplatin.^360^ IGF2BP2 positively modulated glutamine metabolism in AML cells via advancing mRNA stability and translation of several metabolic-related genes, such as GPT2, SLC1A5, and MYC, thereby accelerating AML development.^361^

Recently, Chen et al. have proposed that m6A methylation was involved in WZ35-mediated enhanced radiotherapeutic sensitivity via Glutathione (GSH) exhaustion. Mechanistically, WZ35 consumed GSH through the ROS-YAP-AXL-ALKBH5-GLS2 loop, inducing metabolic remodeling and further repressing GC cell metastasis.^362^ Moreover, METTL16 participated in regulating branched-chain amino acid (BCAA) metabolism by facilitating expression of BCAT1 and BCAT2 in an m6A-dependent manner, serving as an oncogene in leukemogenesis and LSC maintenance of AML.^363^

**Fig. 5:**
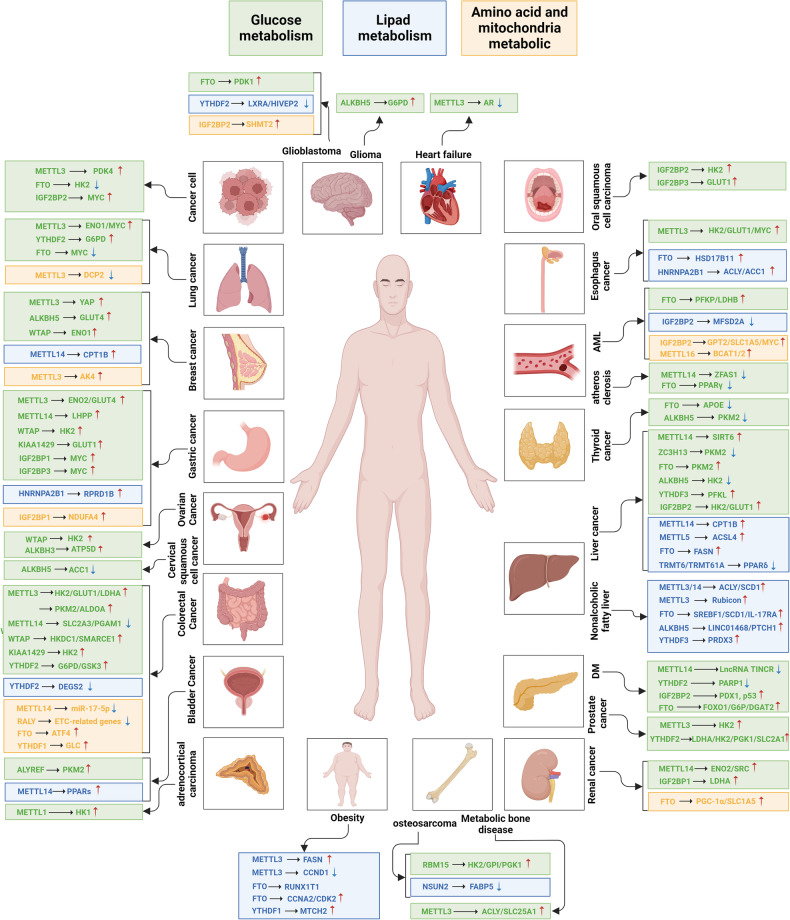
Epigenetic regulation of RNA modification on metabolism in diseases. RNA modifications broadly regulated metabolic pathways in diverse diseases, covering glucose (green box), lipid (blue box), amino acid and mitochondrial (yellow box) metabolism. As intuitively shown in the picture, Gastric cancer (GC), Colorectal Cancer (CRC), Hepatocellular Carcinoma (HCC), Acute Myeloid Leukemia (AML), and Breast Cancer (BRCA) are especially related to RNA modifications-mediated metabolic deregulations. The figure is generated with BioRender (https://biorender.com)

In summary, as shown in Fig. 5, Numerous proteins and RNA modification mediated metabolism are implicated in the progression of various diseases. To put into practice the preventive and treatment options for these diseases, a thorough understanding of RNA modification and metabolic disorders is needed.

## RNA modification and immunometabolism

**Fig. 6:**
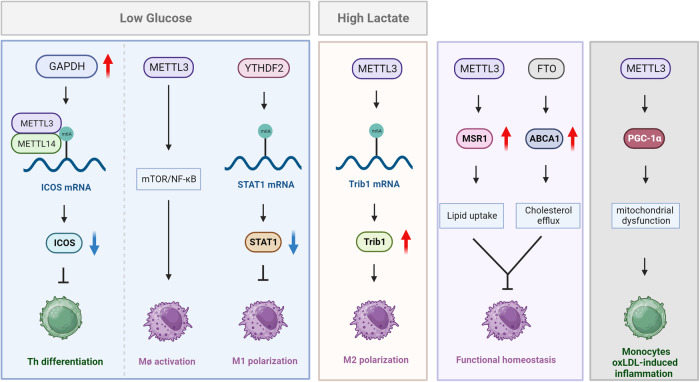
Effects of RNA modification on immunometabolism. This figure shows current findings about how RNA modifications regulate immunometabolism. On the one hand, RNA modifications are involved in the intrinsic metabolic adaptation of immune cells, further affecting function and state of immunocytes. On the other hand, m6A modification could mediate several phenotype alterations of immunocytes induced by glucose deficiency and high lactate in TME. The figure is generated with BioRender (https://biorender.com)

Generally speaking, the immunometabolism concerns distinctions between activated and resting immune cells. The former metabolizes in a manner similar to malignant cells, Warburg effect without obvious OXPHOS, while the latter obtains energy from FAO and the Krebs cycle. Corresponding metabolic patterns of different immune cell subgroups have been described.^364^ Herein, we focus on the contributions of RNA modifications on immunometabolism in diverse immune responses (Fig. 6).

### Anti-tumor immunity

The concept of tumor immune microenvironment (TIME) emphasizes the interplay between immune cells, tumor cells, and other components in immunity system, which profoundly influences the immune responses, via nutrients depletion and metabolites release. To sustain the rapid proliferation, tumor cells consume large amounts of glucose, glutamine and other amino acids like arginine, generally constituting an adverse residential environment for immune cells. The universal recognition is that glucose exhaustion induced by tumor cells contributes to immunosuppressive TME.

Nevertheless, the pioneering work of Reinfeld et al. showed that glucose is not a limiting factor in the TME, and resident immunocytes were capable to enhance glucose uptake in compensation for depleted glutamine. Indeed, multiple pathways are conducive to the impaired glucose metabolism of T cells from TME, but the effects are likely to be context-dependent. Mechanism study about epigenetic regulation on glycolytic reprogramming of T cells remains deficient, but the glycolytic-epigenetic interplay has been found in development of Tfh cells. VHL deficiency induced expression of glycolytic enzyme GAPDH, which acted as an epigenetic regulator to enhance METTL3/METTL14-catalyzed m6A modification on ICOS mRNA, thereby suppressed ICOS expression led to attenuated Tfh cell differentiation.^365^

Glucose depletion in TME predisposes the differentiation of macrophages to M2-like TAM, which preferentially employs OXPHOS for ATP synthesis rather than consuming glucose.^366^ Recently, METTL3 was considered as the top candidates for regulating M1 macrophages activation via targeting mTOR/NF-κB-mediated metabolic adaptation.^367^ However, Ning et al. proposed that m6A modification was responsible for inhibited glycolysis and M1 macrophage polarization, through YTHDF2-mediated degradation of m6A-modified STAT1 mRNA, further attenuating glycolysis-related genes expression.^368^

Distinct glutamine acquisition is another essential aspect of nutrient partitioning. Tumor cells tend to have advantage in glutamine consumption over those immune cells, as tumor cells have over-expressed methionine transporter Slc43a2, thereby restricts methionine metabolism and the antitumor function of T cells.^369^ However, whether RNA modifications participate in glutamine metabolism of immune cells remains to be explored.

Except for nutrient competition, metabolites secreted by tumor cells also exert immunosuppressive effects on anti-tumor immunity. Tumor cells produce large amounts of lactic acid to generate highly acidic regions, as a hallmark of immunosuppressive TME. High concentration of extracellular lactic acid significantly inhibits the survival and activation of T and NK Cells, further blocks the immunosurveillance.^370^ m6A-mediated stabilization of circQSOX1 enhance lactic acid accumulation in CRC, thus supports Treg cells and facilitate immune escape, which further impacts efficacy of anti-CTLA-4 therapy in vivo.^229^ As mentioned above, the intratumoral myeloid cells have vigorous glycolytic activity, but whether myeloid-derived lactate limits T cell effector functions, remains to be explored.^371^

Lactate also negatively influences functions of macrophage and skews the differentiation of macrophages toward the M2 phenotype. M2 macrophage infiltration in endometriosis was positively correlated with lactate accumulation. Mechanism study showed that lactate promoted M2 macrophage polarization via METTL3-mediated m6A modification on Trib1 mRNA, which enhanced its stability.^372^

Tumor cell is a significant source of intratumoral lipids, including cholesterol, fatty acid or oxidized lipids, which have a deleterious effect on T cells, DCs, and macrophages. Several studies have confirmed that m6A methylation was involved in maintaining the functional homeostasis of macrophages via targeting the balance between lipid uptake and cholesterol efflux. Elevated METTL3 in oxidized low-density lipoprotein (oxLDL)-treated macrophages facilitates lipid uptake via interacting with DDX5 to target MSR1 mRNA and stabilize it in m6A-dependent manner.^373^

FTO is upregulated in macrophages loaded with ox-LDL, which enhances cholesterol efflux via motivating AMPK/ACC phosphorylation to promote ABCA1/G1-mediated efflux, and attenuates cholesterol ester accumulation through restricting PPARγ to reduce CD36 expression.^316^ More direct and convincing evidences are expected to elucidate the epigenetic regulation on TAMs. Besides, cholesterol uptake activates PD-1 expression in tumor-infiltrating CD8 + T cells, which in turn facilitates FAO and lipolysis.^374^ Extracellular fatty acids are more effectively consumed by Treg cells than effector T cells, which eventually supports Treg accumulation.^375^ Oxidized lipids restrain cross-representation in DCs^376^ and enhanced uptake of fatty acids and peroxidation lead to dysfunctional state of tumor-derived DCs.^377^ But the role of RNA modifications in these cellular processes has not been identified yet.

### Antiviral immunity

The pathogenesis of infectious diseases is consisted of two parts, deficiency of immune system itself and immune escape of pathogens. On one hand, specific RNA modifications on viral RNAs have been described, including m6A, m5C ac4C, Ψ, and RNA editing, thus affecting viral RNA sensing and signaling.^378^ On the other hand, RNA modifications influence host responses to viral infection via regulating immune cell functions. The interferon pathway is the major target of m6A modification to modulate antiviral innate immunity. METTL3/14 enhances the turnover rate of IFNB mRNAs via YTHDF2-mediated manner and accelerates viral propagation.^379,380^ The coordination between m6A methylation and other RBPs also mediate the negative effects on immunity. DEAD-box helicase 5 (DDX5), hijacked by viruses to promote replication, could interact with METTL3 to facilitate formation of the METTL3/14 complex during vesicular stomatitis virus infection.^381^ In addition to interferon pathway, ALKBH5 could promote viral propagation relying on metabolic rewiring. It was showed that viral infection impaired the enzymatic activity of ALKBH5 in posttranslational level and thus downregulated expression of α-ketoglutarate dehydrogenase (OGDH), leading to reduced itaconate production, a metabolite that inhibits viral replication.^382^ Beyond that, the association between RNA modification and metabolic processes remains largely unknown.

### Inflammation and autoimmune disorders

Inflammatory response is achieved through a coordinately regulated gene expression program, including acute and chronic type.^383^ In response to microorganisms, autoimmunity, allergies, dysregulated metabolism, and physical damage, different types of inflammation are produced.^384^ Until recently, regulatory roles of RNA modification in inflammation and anti-inflammation gene expression have been verified. Previous studies have shown that m6A modification is involved in pathogenesis of autoimmune diseases. For instance, METTL3 is significantly upregulated in RA patients and positively associated with CRP and ESR, the two common markers of RA disease activity.^385^ In systemic lupus erythematosus (SLE), decreased m5C level and low NSUN2 expression are found in CD4 + T cells, and hypermethylated m5C-modified upregulated genes in SLE are enriched in inflammatory pathways.^386^ Significantly, in DC-dependent inflammatory response, m6A-mediated glycolytic reprogramming is critical for feedback-control of DC migration. Mechanistically, in response to microbial products or inflammatory signals, upregulated CC-chemokine receptor 7 (CCR7) stimulated lnc-Dpf3 via removing its m6A modification to prevent degradation, and lnc-Dpf3 could negatively modulate HIF-1α pathway via binding to HIF-1α and suppressing HIF-1α-dependent transcription of the glycolytic gene Ldha.^387^ Also, m6A modification modulates macrophage phenotype in inflammatory responses. Previous study has reported that METTL3 was notably elevated in M1 macrophages and modulated polarization via metabolism reprogramming. In mechanism, m6A methylation contributes to enhanced expression of HDGF, which increases glycolysis and lipids accumulation in M1, therefore aggravating the progression of atherosclerosis.^388^ And METTL3-meidated m6A of PGC-1α mRNA is involved in mitochondrial dysfunction and oxLDL-induced inflammation in monocytes.^340^ Although there are few studies on RNA modifications regulating inflammatory and autoimmune diseases in the aspect of immunometabolism.

## Clinical implications of RNA modifications

### RNA modifications and therapeutic responses of metabolic therapy

For the currently approved metabolic drugs, an impending challenge of clinic application is development of chemo-resistance owing to rewiring or compensatory metabolic pathways. Thus, the multiple pathways blockade or combined therapy may have superiority over the single-agent therapy. Notably, multiple studies have supported that combined utilization of targeting RNA modifications could improve chemo-resistance to some metabolism-targeted drugs.

The influences of m6A modification on CRC resistance to 5-FU is a representative example. Mechanism studies have demonstrated that METTL3 could induce 5-FU resistance of CRC cells via m6A/DGCR8/miR181d/NCALD axis,^389^ m6A/IGF2BP1/SEC62/β-catenin axis.^390^ Also, m6A methylation facilitates preferential splicing of p53 pre-mRNA to produce p53 R273H mutant protein, leading to multidrug resistance in CRC cells.^391^ Moreover, suppressing c-Myc-driven YTHDF1 transactivation was revealed to re-sensitize CRC cells to some anticancer drugs, including 5-FU.^392^ Consistently, Jiang et al. found that miR-136-5p could downregulated YTHDF1 to suppress tumor progression and chemoresistance to 5-FU, while miR-136-5p was declined in CRC cell lines and tissues.^393^

Moreover, METTL3 was identified to positively modulate gemcitabine (GEM) sensitivity of PC via DBH-AS1/miR-3163/USP44, and low expression level of METTL3 was closely related with GEM resistance.^394^ Upregulated METTL14 was observed in GEM-resistant PC cells, which was induced by p65 and downstream facilitated cytidine deaminase (CDA) expression to inactivate GEM in PC. Inhibition of METTL14 effectively re-sensitized GEM in vitro and in vivo, indicating a promising approach for circumvent chemo-resistance.^395^ Intriguingly, ALKBH5-mediated demethylation also exerts a positive role in GEM sensitivity of pancreatic ductal adenocarcinoma (PDAC) through suppressing Wnt pathway.^396^

### RNA modifications and therapeutic responses of immunotherapy

Growing researches revealed that m6A regulators markedly affected therapeutic responses against checkpoint blockade. Wang et al. reported that depletion of METTL3/14 enhanced infiltration and cytokines secretion of CTL, augmenting anti-PD-1 therapy efficacy of CRC in vivo, through m6A/YTHDF2/STAT1/IRF1 axis.^397^ However, METTL14 could sensitize cholangiocarcinoma to ICB via YTHDF1-mediated degradation of SIAH2 mRNA.^398^ Knockdown of YTHDF1 enhances cross-presentation of DCs to CD8 + T cells by suppressing cathepsins expression, further increased IFN-γ secretion of T cells upregulates PD-L1 level in tumor cells.^399^ FTO was identified to negatively regulate ICB therapeutic efficacy in melanoma. Ablating FTO decreases expression of several significant melanoma-promoting genes and sensitized anti-PD-1 treatment in vivo.^400^ However, FTO was revealed to promote PD-L1 expression in an IFN-γ-independent manner of CRC cells, thus improving ICB treatment.^401^ Su et al. demonstrated that inhibition of FTO obviously downregulated immune check point gene LILRB4 in AML cells, with superiority over PD-L1/2, further repressing leukemia stem cell maintenance and immune evasion.^402^ In melanoma, FTO participates in rewiring tumor glycolysis metabolism to suppress T cell effector functions, and FTO inhibition synergizes with anti-PD-L1 therapy.^248^ Additionally, the silico analysis identified that high FTO level was associated with poor prognosis and unfavorable immunotherapy effect of GC patients.^403^ And ALKBH5 was supposed to be a potential predictor for anti-PD-1 blockade efficacy in melanoma. Deficiency of ALKBH5 induced downregulation of MCT4 expression and intra-tumoral lactate content, which negatively influenced polymorphonuclear myeloid derived cells and Tregs.^404^ Several bioinformatic studies have underlined the intimate connection between m6A modification and immunotherapy resistance.^405^ Moreover, loss of A-to-I editor ADAR1 significantly augmented anti-PD-1 treatment in melanoma and CRC. In mechanism, the interaction of tumor intrinsic type I and type II IFN signaling contribute to sensitize ADAR1-null cells to ICB.^406^

Except for checkpoint blockade therapy, targeting m6A modification has demonstrated promising potential in improving adoptive cell therapy. Marvelous progresses have been made in modulating METTL3 and YTHDF2 to enhance the proliferation and cytotoxicity of NK cells in vitro, which might inspire future protocols for NK cell-based immunotherapy.^407,408^ No attempt to modulate m6A methylation in CAR T cells has been reported yet, but considering the significant roles of m6A regulators in determining functions and fate of T cell, novel therapeutic strategies are expected.

### Development of RNA modification-targeted agents

Targeting the dysregulated m6A regulators, which are overexpressed in tumor on most occasions, plentiful specific inhibitors have demonstrated exciting anti-tumor effects in vitro and in vivo (Table 6). FTO is considered as the most promising target. Within a decade, a series of selective inhibitors have come out, ranging from natural substance to small-molecule compound. The first natural inhibitor Rhein displayed therapeutic activity in leukemia mice,^409^ meclofenamic acid 2 (MA2) was observed to suppress glioblatoma progression.^410^ Small-molecule compounds CHTB and N-CDPCB were identified with novel binding sites by crystal structure screening.^411^ R-2-hydroxyglutarate (R-2HG) exerts anti-leukemia and anti-glioma effects, synergizing with current first-line chemotherapy agents.^412^ FB23-2 also significantly attenuates the progression of AML in vitro and in xeno-transplanted mice.^413^

**Table 6:** Specific inhibitors against RNA modification regulators

Target	Drug	Cancer	Effect	Ref.
FTO	Rhein	AML	Anti-leukemia efficacy in vitro and in vivo	^409^
	MA2	GBM	Suppresses cell proliferation and tumor progression	^410^
	R-2HG	AML/GBM	Antitumor effect, synergizing with first-line chemotherapy agents	^412^
	FB23-2	AML	Anti-leukemia efficacy in vitro and in vivo	^413^
	CS1/2	AML	Potent anti-leukemia efficacy in mouse models, sensitize leukemia cells to T-cell cytotoxicity, overcomes immune evasion	^402^
	Dac15	Melanoma	Promotes activation and effector state of T cell, improving anti-PD1 blockade	^248^
	18097	BRCA	Restrain in vivo growth and lung colonization	^414^
	FTO-43	GC/AML/GBM	Potent anti-tumor effects in mouse model	^415^
	C6	ESCC	Anti-tumor efficacy in vitro and in vivo	^416^
ALKBH5	ALK-04	Melanoma	Improve anti-PD-1 therapy efficiency	^404^
	Compound 1/2	AML	Anti-proliferative effects in specific AML cell lines	^417^
METTL3	UZH1a	AML	Suppress proliferation and viability of tumor cells	^418^
	UZH2	AML/PC	More potent anti-proliferative effects in vitro	^419^
	STM2457	AML	Anti-leukemia efficacy in vitro and in vivo	^420^
TRMT6/TRMT61A	Thiram	HCC	Suppresses HCC growth in preclinical models	^421^

Subsequently, more potent inhibitors with potential to improve anti-tumor immunity was developed. CS1/2-induced FTO inhibition not only attenuates leukemia stem cell self-renewal, but reprograms immune responses by downregulating expression of immune checkpoint gene, which overcomes HMA-induced immune evasion and sensitizes leukemia cells to T cell cytotoxicity.^402^ Also, Dac15 restores functions of CD8 + T cells, blocks FTO-mediated immune evasion, and synergizes with anti-PD-1 blockade.^248^ Recently, progresses have been made in more tumor types rather than AML and glioma. A small molecule inhibitor 18097 significantly restrained in vivo growth and lung colonization of breast cancer cells.^414^ Oxetanyl class demonstrated antiproliferative effects in GC, glioblastoma and AML models, while FTO-43 has potency comparable to 5-FU.^415^ Compound C6, a 1,2,3-triazole analogs, was suggested as a potential orally antitumor agent for esophageal cancer.^416^

Exploitation of inhibitors against other regulators, including METTL3 and ALKBH5, is also proceeding steadily. The ALKBH5 inhibitor ALK-04 effectively sensitized melanoma cells to anti-PD-1 blockade, as ALKBH5 attenuated immunotherapy responses via regulating lactate content and immunosuppressive cell infiltration in the TME.^404^ Selberg et al. discovred two compouds, 2-[1-hydroxy-2-oxo-2-phenylethyl]sulfanyl acetic acid and 4-[furan-2-yl]methyl amino-1,2-diazinane-3,6-dione, demonstrated cancer-cell-type-selective antiproliferative effects in selected leukemia cell lines.^417^ Targeting SAM binding sites, adenosine was first identified as METTL3 inhibitors. Non-nucleoside inhibitors with higher selectivity and permeability have been developed, such as UZH1a and UZH2.^418,419^ A novel METTL3 inhibitor STM2457 effectively blocked AML progression and prolonged survival in AML mouse models, without disturbing normal hematopoiesis.^420^

Furthermore, RNA m1A methylation is also a potential therapeutic target. The m1A methyltransferase complex, TRMT6/TRMT61A is highly expressed in HCC and correlated with poor prognosis. Wang et al. screened out three potential drugs targeting the interaction of TRMT6 and TRMT61A, thimerosal, phenylmercuric acetate (PMA), and thiram. Among them, the administration of thiram significantly attenuated HCC growth in preclinical models.^421^

### RNA modifications in RNA-based therapeutics

Though RNA medicine has been facing challenges like efficacy and immunogenicity since from birth, the most recent hit of mRNA vaccines against COVID-19 provide new momentum to this field and bring RNA modifications back into to spotlight. Chemical modification of RNA could protect RNA from hydrolysis and nucleases, and decrease off-target cytotoxic effects. Once therapeutic RNAs form duplexes with targeted sequences, modifications lowering the melting temperature could destabilize the complex and improve target specificity via reducing base-paring with non-target RNAs. Moreover, RNA modifications are utilized for RNA delivery and strengthen the pharmaceutical activity of RNA.^422^

Base modifications have been successfully applied in improving the performance of therapeutic RNA, for example, replacement of uridine with the modified base 1-methylpseudouridine (N1-Me) in COVID-19 vaccines (Pfizer’s Comirnaty and Moderna’s Spikevac) effectively facilitates translation and reduces off-target side effects and immunogenicity of therapeutic mRNA.^423^ Additionally, m7G cap linked by a 5′-triphosphate to the 5′ end of the mRNA, which replicates the naturally occurring mRNA caps to prevent degradation of the 5′ end of mRNA, has been introduced into mRNA vaccines BNT162/Comirnaty and mRNA-1273/Spikevax.^424^ As for ribose modifications, modified hydroxyl group on the C-2′ position of the ribose could protect RNA against nuclease digestion and lower the thermal stability of duplexes. N-acetylgalactosamine (GalNAc) groups or lipophilic moieties attached cleavable linkers, including ester-based, peptide-based cleavable groups, could localize therapeutic RNA to target tissue.^425^ Moreover, modifications to the phosphate group in the sugar-phosphate backbone shelter RNAs from nucleases, represented by phosphorothioate. And eliminating the negative charge via replacing the oxygens on the phosphates with neutral groups or cations can assist the delivery into cell.^426^

Based on CAS Content Collection, a recent study summarized the modification content in approved RNA medicines, including antisense oligonucleotide (ASO), siRNA, aptamer, and mRNA.^427^ Thereinto, N1-Me is prominently abundant in two mRNA vaccines, along with 2′-O-methyl, 3′-methyl, m7G, 5′-5′-triphosphate. And 2′ -oxy-methoxyethylguanosine (2′-MOE) is exclusive in ASOs, which protects ASOs from degradation. The approved siRNAs have 2′-fluoro and 2′-O-methyl modification of the ribose, and three of them are 3′-glycosylated with the GalNAc conjugate, which specifically targets siRNAs to hepatocytes.^428^

### Combination of targeting RNA modification and current therapy

Some gratifying results have been acquired in combined application of m6A regulators inhibitors with current anticancer therapy. The involvement of m6A modification in underlying mechanisms of resistance has been systematically summarized.^429^ Herein, we put emphasis on the combined utility of targeting RNA modification to circumvent resistance and improve individualized cancer treatment.

A mass of evidences showed that overexpressed METTL3 widely participated in the acquisition of various therapeutic resistance in many cancer types. Knockdown of METTL3 using short hairpin RNA improved sensitivity to anticancer reagents such as gemcitabine, 5-fluorouracil, cisplatin and irradiation in pancreas cancer (PC).^430^ Suppression of METTL3 restored chemosensitivity and attenuated CML cells viability.^431^ Nevertheless, few studies have ever investigated the utility of METTL3 inhibitors in overcoming chemoresistance. Targeting FTO also shed new light on improving chemoresistance. Upregulated FTO in oral squamous cell carcinoma played a pivotal part in arecoline-induced stemness and chemoresistance to cisplatin.^432^ Depletion of FTO sensitized breast cancer to doxorubicin via suppressing de novo synthesis of fatty acid.^433^ Specifically, FTO was revealed to facilitate GBM resistance to temozolomide (TMZ), and the inhibitor R-2HG demonstrated a synergistic effect with TMZ in suppressing proliferation of FTO-high glioma cells.^412^

Moreover, the feasibility of administrating m6A regulators inhibitors to improve immunotherapy effectiveness needs further investigation. Depletion of METTL3/14 was found to augment ICB therapeutic responses in mismatch-repair-proficient or microsatellite instability-low (pMMR-MSI-L) CRC and melanoma.^434^ In accordance, a recent study confirmed that targeting METTL3 by inhibitor STM2457 potentiate ICB efficacy in various CRC mouse models.^435^ Knockdown of FTO reduced PD1 expression in melanoma via m6A/YTHDF2-dependent manner, thus sensitized anti-PD-1 blockade.^400^ FTO was supposed to enhanced PD-L1 expression independent on IFN-γ in CRC.^436^ In AML, FTO inhibition induced by small-molecule compounds CS1/2 leads to downregulation of checkpoint gene LILRB4, reigniting the interest of introducing ICB to AML.^402^ Besides, Li et al. reported that the specific inhibitor to ALKBH5, ALK-04 markedly enhanced the efficacy of anti-PD-1 blockade in CRC model.^404^

### Clinical trials targeting RNA modifications

We surveyed ClinicalTrials.gov as of October 20, 2023, to keep up-to-date with clinical implications of RNA modifications, basically including therapeutic effectiveness of agents targeting modifiers, potential as predictive biomarkers, and combined application with current treatment. However, hardly any above-mentioned specific inhibitor has progressed into clinical stage, in spite of the encouraging antitumor results of FTO-targeted agents in various cancers. Most recently, a phase 1, first-in-human study is designed to systematically evaluate the pharmacokinetics, pharmacodynamics and clinical activity of STC-15 in adult subjects with advanced malignancies (NCT05584111).

Several studies have evaluated the association between FTO polymorphisms (rs9939609 and rs1558902) and obesity in different populations, including Turkish population (NCT04205318), Indonesian obesity women (NCT04740528), as well as weight loss in overweight carriers induced by calorie restriction (NCT02940197), and intermittent or moderate continuous high intensity training programs (NCT03568773). Furthermore, there are projects aim at assess the correlation between FTO polymorphisms and risk of developing diabetes in Mexican adolescents with overweight and obesity (NCT02886013), and features of metabolic syndrome in children with T1D (NCT01279161). Considering that variants in FTO showed high correlation with body weight and also interact with dopamine signaling in the brain, a clinical trial was designed to develop a genotype-specific and individualized therapy approach for obesity targeting FTO (rs8050136) variant (NCT03525002). Genotyping for FTO was also incorporated into tailored therapeutic model for azathioprine-induced myelosuppression in inflammatory bowel disease patients (NCT03719118).

## Conclusion and future perspectives

### State-of-the-art methods for RNA modification sequencing

With advent of high-throughput methodologies, precision and sensitivity of RNA modification sequencing invented in an unprecedented space. Currently, mainstream MeRIP- and miCLIP-based methods have been widely accepted, yet with several disadvantages to be overcome. The poor sensitivity of antibody-based methods is first limitation, and chemical-assisted labeling is recognized as a promising approach. On account of the strong affinity of biotin-streptavidin binding, the m6A seal (m6A selective chemical labeling) method dramatically enhance enrichment efficiency via introducing a biotin tag to modified bases.^437^ In addition, to solve the incapability of quantifying modification ratio, m^6^A-LAIC-seq (m^6^A-level and isoform-characterization sequencing), originated from MeRIP-seq, could quantify m6A levels for all isoforms of transcripts for each gene via isolating m6A-positive and m6A-negative post-RIP fractions and sequencing full-length transcripts.^438^ Adding synthetic modification-free RNA molecules as internal reference is another strategy to realize quantitative sequencing.^439^ To be noted, single-cell sequencing technologies is an emerging hotspot in tumor immunology, which could effectively profile the intricate immune landscape in tumor TME. For instance, DART-seq (deamination adjacent to RNA modification target sequencing) is designed to monitor m6A at the single-cell level, which successfully reveal the heterogeneity in m6A scenarios across individual cells and identify differentially methylated mRNAs across the cell cycle.^440^ However, further application of DART-seq in clinics is limited by its dependency on overexpression of the APOBEC1-YTH fusion protein in cells. Hence, a free of genetic manipulation single-cell method for deciphering RNA modification is warranted.

### Advancement of RNA modification databases

With advanced methodologies of detecting and profiling RNA modifications, rapid-accumulated enormous epi-transcriptome data call for centralized bioinformatics platforms to mine the underestimated treasure. For both experimental and computational studies of RNA modifications, such valuable resources will be of great help. Researchers focus on structural biology could take full advantage of comprehensive databases like RNAMDB and MODOMICS, while computational biologists perform their researches based on relational databases such as MetDBv2.0, m6A-Atlas, RMBase v2.0 etc. Exploration of novel types or modifiers of RNA modification fully relies on current knowledgebases. For clinics, these databases advance understanding of the role epi-transcriptomics plays in disease pathology. Not a few databases have provided information about relationship between disease-related varients with RNA modifications, such as m6A-Atlas, RMVar, and RMDisease.

Meanwhile, epitranscriptomics-disease links are highlighted in newest update of MODOMICS. The new section exhibits association between malfunction or misregulation of a given RNA-modifying enzymes with specific disease conditions. To better elucidate the post-transcriptional regulatory networks, multi-omics analysis is highly rated. Moreover, context-specificity of RNA modification should be taken into consideration via distinguishing species, cell type, and tissues. Finally, more user-friendly interface and webserver tools are significant for improving accessibility of these resources.

### RNA modifications and immunology

During differentiation and development of immune cells, various clusters of functionally coordinated genes are under sophisticated control of RNA modifications. The highly selectivity and specificity of RNA modifying machinery still remains largely defined. The top priority is to distinguish different targeted transcripts according to a framework of classical immunological systems such as polarization of macrophages and CD4 + T cell differentiation. It was suggested that additional factors such as region-enriched cis-regulatory elements exerted a certain effect on selectivity of RNA marking. Besides, increasing evidences have implicated the crosstalk between RNA modifications on non-coding chromosome-associated regulatory RNA (carRNA) and chromatin modifications, thus RNA modifications may control immune responses to environmental stimuli via shaping the chromatin environment of immune cells.

In the aspect of tumor immunology, the discrepancy of epi-transcriptome between tumor and immune cells is acknowledged as an essential influencing factor of antitumor immune responses. However, relevant research is still in its infancy. Thus, rigorous dissection of RNA modification marks and regulators in tumor cells and immune cells is considered as a fundamental and crucial for developing effective interventions. Following marker-informed sorting of cell populations of interest, methods like mass spectrometry are used for profiling dynamic RNA modifications. Furthermore, integration of single-cell scale and transcriptome methods with RNA modification sequencing may provide valuable insights into dysregulated RNA modification in the TME. Aside from highly-specific RNA modification-targeted inhibitors, modification editing in immune cells is another promising direction for treating immune-related diseases.

Established on the understanding of metabolism, the application prospects of targeting m6A methylation in immunotherapy mainly consisted of two possibilities. One is to circumvent therapeutic resistance mediated by the metabolic antagonism in TME, the other is to potentiate proliferation efficiency and effector functions of immune cells for adoptive cell therapy. Recent advances clued some potential strategies: 1) a programmable m6A editing machinery to fine-tune RNA modifications of specific genes with minimal off-target alterations, 2) effective manners to manipulate m6A system ex vivo for optimal generation of NK cells and T cells, 3) efficient targeted delivery of m6A editors into cells, like nanoparticles, 4) inhibitors against m6A regulators with potential to modulate anti-tumor immunity.

### RNA modifications and cancer metabolism, metabolic diseases

The current understanding is that metabolic phenotypes evolve as cancers process from premalignant lesions, localized invasive malignancies to metastatic cancers, even therapy-resistant states. The dynamic RNA modification along with emerging metabolic vulnerabilities in evolutionary process provide attractive clinical opportunities. In some cases, tumors exhibited stereotyped metabolic alterations without detectable mutations or DNA methylation abnormities,^441^ implicating the presence of other epigenetic regulation like RNA modifications. Delineating the evolving genetic, epigenetic, immune-metabolic landscape is quite necessary for designing effective strategies to preclude metastasis. Progresses in spatial-omics techniques and system biology research may help to address it.

Hyperactive metabolic pathways lead to brisk adaptation to nutrient deprivation, contributing to resistance to antimetabolic chemotherapy agents like antifolates. Metabolic coupling, characterized by catabolites transfer, is common in tumor for overcoming nutrient deficiency. Thus, combination of targeting glycolysis and OXPHOS was proposed as a promising strategy. Considering the underlying toxicity, the alternative is suppressing dysfunctional signals to indirectly target glycolysis, while directly targeting OXPHOS.

Increasing studies have indicated the significance of epigenetic regulation in metabolic diseases. Up to now, none of epigenetic drugs have been approved for metabolic diseases, and the efficacy of RNA modification-targeted agents have not been verified in metabolic diseases. Thus, investigation whether inhibition of RNA modifiers can be used for treatment of metabolic diseases is requested. Given that environmental factors shed influences on epi-transcriptome via intracellular metabolic changes, molecular insights of RNA modification in development of metabolic diseases remains largely unknown.

### Superiority and challenges of targeting RNA modifications

Indeed, the universal distribution and broad functionality of RNA modification is a double-edged sword. For anticancer treatment, targeting a single identified driver sometimes turns out an unfavorable result, as a consequence of various reasons including the development of resistance and intra- or intertumoral heterogeneity. From this point, targeting RNA modification is advantageous to cover a network of targets. Especially, these RNA modifiers tend to be overexpressed or more active in cancerous tissue compared to matched normal control tissue. However, the essentiality and specificity of these RNA modifiers remain significant concerns.

For currently developed agents targeting RNA modifying enzymes, poor specificity and selectivity remain the main obstacle in their progression into clinical researches. And such deficiency is anticipated to be improved via optimized bioinformatic prediction models and high-throughput enzymatic tests. Here are several other outstanding questions to be further investigated. If pharmacologic inhibition of RNA modification enzyme is capable to reproduce the phenotypic activity induced by genetic deletion? If redundance in modifying enzymes, like METTL3/14 complex and METTL6, potentially induce resistance to pharmacological inhibition. Given that RNA modifiers tend to be overexpressed in tumor tissue but still present in normal tissues, an appropriate therapeutic window in a certain therapeutic context may be necessary. Aside from specific inhibitors or activators to those modifiers, RNA modifications have been applied to improve the stability, efficacy and target specificity of RNA-based therapies. Common strategies include utilizing synthetic chemical or naturally occurring modifications, and modulating sequence context or location of these modifications. For all current therapeutic RNA, RNA modifications are extensively present and poised to further enhance their effectiveness.

In summary, epigenetic regulation of RNA modifications exerts a crucial role in cellular metabolism in diverse physiological and pathological situations. Growing evidences suggest that such metabolic-epigenetic interplay significantly affects immune responses, via modulating biological activities of immune cells and remodeling immune context. Thus, delineating the evolving genetic, epigenetic, immune-metabolic landscape is quite necessary for designing effective strategies to preclude pathogenesis, including various metabolic disorders, immune-related diseases, and cancer. Recent years have witnessed remarkable advancements in methods for detecting and profiling RNA modifications, accompanied with a series of serviceable databases and tools springing up. At present, attempts to targeting RNA modification for improving current therapy have obtained some inspiring advances, but relevant researches are still in its infancy. And we can count on further in-depth exploration to accelerate the development of RNA modification-targeted therapy, metabolism-targeted therapy and immunotherapy.
